# Effect on Postpartum Hemorrhage of Prophylactic Oxytocin (10 IU) by Injection by Community Health Officers in Ghana: A Community-Based, Cluster-Randomized Trial

**DOI:** 10.1371/journal.pmed.1001524

**Published:** 2013-10-01

**Authors:** Cynthia K. Stanton, Samuel Newton, Luke C. Mullany, Patience Cofie, Charlotte Tawiah Agyemang, Edward Adiibokah, Seeba Amenga-Etego, Niamh Darcy, Sadaf Khan, Deborah Armbruster, John Gyapong, Seth Owusu-Agyei

**Affiliations:** 1 Johns Hopkins Bloomberg School of Public Health, Baltimore, Maryland, United States of America; 2 Kintampo Health Research Centre, Ghana Health Service, Kintampo, Ghana; 3 PATH, Ghana, Accra, Ghana; 4 Research Triangle Institute, North Carolina, United States of America; 5 PATH, Seattle, Washington, United States of America; 6 United States Agency for International Development, Washington (DC), United States of America; 7 School of Public Health, University of Ghana, Accra, Ghana; The University of Adelaide, Australia

## Abstract

**Background:**

Oxytocin (10 IU) is the drug of choice for prevention of postpartum hemorrhage (PPH). Its use has generally been restricted to medically trained staff in health facilities. We assessed the effectiveness, safety, and feasibility of PPH prevention using oxytocin injected by peripheral health care providers without midwifery skills at home births.

**Methods and Findings:**

This community-based, cluster-randomized trial was conducted in four rural districts in Ghana. We randomly allocated 54 community health officers (stratified on district and catchment area distance to a health facility: ≥10 km versus <10 km) to intervention (one injection of oxytocin [10 IU] one minute after birth) and control (no provision of prophylactic oxytocin) arms. Births attended by a community health officer constituted a cluster. Our primary outcome was PPH, using multiple definitions; (PPH-1) blood loss ≥500 mL; (PPH-2) PPH-1 plus women who received early treatment for PPH; and (PPH-3) PPH-2 plus any other women referred to hospital for postpartum bleeding. Unsafe practice is defined as oxytocin use before delivery of the baby. We enrolled 689 and 897 women, respectively, into oxytocin and control arms of the trial from April 2011 to November 2012. In oxytocin and control arms, respectively, PPH-1 rates were 2.6% versus 5.5% (RR: 0.49; 95% CI: 0.27–0.88); PPH-2 rates were 3.8% versus 10.8% (RR: 0.35; 95% CI: 0.18–0.63), and PPH-3 rates were similar to those of PPH-2. Compared to women in control clusters, those in the intervention clusters lost 45.1 mL (17.7–72.6) less blood. There were no cases of oxytocin use before delivery of the baby and no major adverse events requiring notification of the institutional review boards. Limitations include an unblinded trial and imbalanced numbers of participants, favoring controls.

**Conclusion:**

Maternal health care planners can consider adapting this model to extend the use of oxytocin into peripheral settings including, in some contexts, home births.

**Trial registration:**

ClinicalTrials.gov NCT01108289

*Please see later in the article for the Editors' Summary*

## Abstract 2

Cynthia Stanton and colleagues conducted a cluster-randomized controlled trial in rural Ghana to assess whether oxytocin given by injection by community health officers at home births was a feasible and safe option in preventing postpartum hemorrhage.

*Please see later in the article for the Editors' Summary*

## Editors' Summary

**Background:**

Many women in low-and middle-income countries die unnecessarily during childbirth, even though the solutions to prevent or manage complications are well known. Maternal death rates are highest amongst poor women living in remote areas, as they are least likely to have access to adequate health care. One of the United Nation's Millennium Development Goals is to reduce maternal death rates by three-quarters by 2015. Between 1990 and 2010, these rates were nearly halved. So there is still some way to go to meet the target.

One of the major causes of maternal death is excessive bleeding after birth, known as postpartum hemorrhage (PPH). The highest rates of PPH are found in Africa (28% of births), with an overall global rate of 11%. PPH can be caused by the uterus not contracting after the baby is born, damage to tissues and blood vessels, retention of the placenta, and problems with blood-clotting.

PPH can be prevented by an injection of oxytocin (a hormone) or with tablets of the drug misoprostol immediately after birth. Other drugs exist but are used much less frequently in low-income countries. If the mother does bleed excessively, then these interventions can also be used to treat PPH in the hours following birth. These drugs cause the uterus to contract. Continued severe bleeding requires emergency treatment in hospital. The World Health Organization (WHO) recommends that in situations where women give birth without the assistance of a trained midwife, priority should be given to preventing PPH because access to emergency services may be limited.

**Why Was This Study Done?:**

Of the two most common options for preventing PPH, oxytocin is generally the preferred choice. It has the advantage of having no side effects, whereas misoprostol can cause fever and shivering. A repeat injection of oxytocin can also be given if the mother continues to bleed excessively, whereas a dose of misoprostol after birth should only be given once. A major concern about both drugs is that the timing of administration must be precise. Giving a drug that causes the uterus to contract before birth can be harmful to both mother and baby. A disadvantage of oxytocin is that it requires someone trained and authorized to give an injection. For this reason, oxytocin has so far been generally limited to hospitals and clinics, where it can be administered by medically trained professionals. Another disadvantage is that oxytocin is weakened by heat, which means its storage and use may be impractical in hot countries.

The main aim of this study was therefore to find out whether health workers without midwifery skills are able to administer oxytocin safely when attending home births in poor, rural communities.

**What Did the Researchers Do and Find?:**

The researchers carried out a cluster-randomized controlled trial in four rural districts in Ghana, working with community health officers (CHOs). CHOs are trained for two years in giving childhood immunizations and antenatal and postnatal care, but are not trained midwives. 54 CHOs were randomized to one of two groups. The CHOs in the first group gave a preventative oxytocin injection to the mother at every birth they attended. The oxytocin was administered using a pre-filled, disposable device called Uniject that is easier and quicker to use than a syringe and needle. The packaging also included a heat-sensitive label that indicated whether the oxytocin still met the manufacturer's criteria for an acceptably potent drug. CHOs in the second group acted as controls, and did not give any oxytocin injections to prevent PPH. The women seen by each CHO formed a cluster. Comparisons were made across the clusters of women that either received or did not receive the preventative intervention.

The researchers found that the women who were given a preventative oxytocin injection lost less blood after birth than the women who did not receive a preventative injection. There were also fewer cases of PPH amongst the women who received oxytocin for PPH prevention. 2.6% of the women who received a preventative oxytocin injection experienced PPH, compared to 5.5% of the controls. Therefore the risk of PPH was approximately halved. There were no cases of oxytocin use before delivery of the baby and no difference in the frequency of other birth complications between women in the intervention and control groups.

**What Do These Findings Mean?:**

These findings show that under the trial conditions, CHOs can safely administer oxytocin injections when attending home births in poor, rural settings. This intervention also proved practical to use in the Uniject format.

The study therefore suggests that oxytocin should be considered for use in regions where maternal deaths from PPH are still unacceptably high. There are also several noteworthy limitations, such as unblinding and the imbalance between participant groups. The researchers emphasized that their findings do not mean that oxytocin is always a better choice than misoprostol for home births. Many factors will influence which intervention is the most feasible, such as the local availability of sufficiently skilled health professionals, the relative cost and availability of the two drugs, as well as ease of access to emergency health services.

**Additional Information:**

Please access these websites via the online version of this summary at http://dx.doi.org/10.1371/journal.pmed.1001524.

- This study is further discussed in a *PLOS Medicine* Perspective by João Paulo Souza
- WHO Factsheet on Maternal Mortality
- United Nations Population Fund's Goals to improve sexual and reproductive health

## Introduction

Hemorrhage is the leading cause of maternal death in low-income countries, estimated to be responsible for one-third of such deaths annually [1]. Postpartum hemorrhage (PPH) is generally accepted to represent the majority of hemorrhage cases and to be predominantly caused by uterine atony. Maternal deaths from hemorrhage in sub-Saharan Africa account for one in five of all maternal deaths world-wide. In sub-Saharan African countries, rates of home birth remain high (decreasing only slowly from 64% in 1995 to 50% in 2008 [2]–[4]), and it is at home births where the consequences of PPH are most severe given the need for immediate treatment of PPH.

Consequently, prevention and treatment of PPH have been the subject of a large body of research over the past decade, prompting the World Health Organization (WHO) to issue a series of evidence-informed guidelines for strategic program planning [5]–[7]. The most recent WHO guideline on PPH prevention recommends the use of a uterotonic drug without other components of active management of the third stage of labor, particularly where skilled birth attendants are not available. In such settings, the use of intramuscular oxytocin (10 IU) via either syringe or a compact, pre-filled, auto-disposable device by an auxiliary nurse, or oral misoprostol (600 mcg) by either an auxiliary nurse or lay health worker trained in the use of misoprostol, is recommended [8]. To date, WHO continues to recommend oxytocin as the drug of choice for PPH prevention at home and in health facilities due to its effectiveness and lack of side effects. Although other uterotonic drugs are listed as alternative drugs, WHO guidelines state “that these alternative drugs should not detract from the goal of widely accessible oxytocin” [7]. Results from a Cochrane meta-analysis of six trials in high-income-country health facilities showed that prophylactic oxytocin (using intramuscular and intravenous dosages from 3 to 10 IU) was associated with a relative risk of 0·50 for PPH (≥500 mL blood loss; 95% CI: 0·43–0·59) and 0·61 for severe PPH (≥1000 mL blood loss; 95% CI: 0·44–0·87) relative to women receiving no uterotonics during the third stage of labor [9].

Research supporting community-based PPH prevention includes three community-based randomized trials in India, Pakistan, and Guinea Bissau that assessed the use of misoprostol (600 mcg) and found protective effects of varying magnitude. PPH was reduced by 47% in the rural India trial where auxiliary nurse midwives attending births in rural health centers administered oral 600 mcg tablets of misoprostol versus placebo (RR: 0.53; 95% CI: 0.39–0.74) [10] and by 24% in rural Pakistan where traditional birth attendants administered oral misoprostol (600 mcg) versus placebo at home births (RR: 0.76; 95% CI 0.59–0.97) [11]. Hoj and colleagues reported an 11% non-significant reduction in risk of PPH for midwife-assisted births at a health center in Bissau, Guinea Bissau (RR: 0.89; 95% CI: 0.76–1.04) [12]. The trials in India and Guinea Bissau also found significant decreases in the risk of severe PPH (blood loss ≥1000 mL); for example, the risk in India was 0.20 (95% CI: 0.04–0.91) [10] and in Guinea Bissau the risk was 0.66 (95% CI: 0.45–0.98) [12]. In all three trials, misoprostol was significantly associated with side effects (shivering and/or fever). To our knowledge, this is the first paper in the literature to describe a randomized trial of prophylactic oxytocin at home births.

The ongoing challenge is to determine the effectiveness and safety of uterotonic drug options and to identify the contexts in which their advantages can be exploited and their constraints overcome. For example, there are three main concerns regarding use of community-based prophylactic oxytocin by unskilled attendants: (1) such providers are often not authorized nor trained to administer injections; (2) potential administration of oxytocin before delivery of the baby to speed labor, which when medically non-indicated and/or when performed without adequate medical supervision and infrastructure can be detrimental to the fetus and the woman [13]; and (3) oxytocin is heat labile [14]. However, an important characteristic of oxytocin is that there are no contraindications for administering an additional 10 IU of oxytocin as an early treatment in women experiencing PPH following prophylaxis with oxytocin. The recommended practice for PPH treatment in health facilities is—where available—intravenous oxytocin, but intramuscular use is not contraindicated as a first response before seeking a higher level of care [7]. In contrast, a key advantage to misoprostol for PPH prophylaxis is that it is heat stable (though requires appropriate manufacturer packaging to avoid degradation from humidity), an important advantage for peripheral settings with difficult access to a health facility. However, given that half or more of PPH cases will continue to occur where misoprostol is used prophylactically, women's only option for treatment is fundal massage and referral since current concerns regarding toxicity prevent misoprostol administration for PPH treatment to women who have received it for prophylaxis [7]. Thus, the women in greatest need, those for whom prophylaxis has been ineffective, must rely on referral and transport from a peripheral site. This limitation is rarely discussed; the India and Pakistan community-based prevention trials provide limited information on successful referral and transport for women experiencing PPH. Another point rarely discussed publicly is the potential use of misoprostol as an abortifacient, which prevents its inclusion in the national drug policies of some countries. Oxytocin is a well-known medicine without this issue.

WHO and others have specifically called for research to determine the safety of prophylactic oxytocin use with an unskilled birth attendant at home births in low-income countries [7],[8]. The trial reported here is designed to assess the effectiveness of a mode of service delivery; that is, can the evidence-based practice of using oxytocin for PPH prevention be effectively delivered by a peripheral health care cadre at home births? We aim to assess whether this mode of service delivery can (a) achieve a health benefit *and* (b) be delivered safely *and* (c) be feasible from a practical standpoint. PPH is the primary outcome of this study for two reasons: (1) there is general consensus regarding the outcome (≥500 mLs of blood loss constitutes PPH) and (2) validated tools exist for the measurement of blood loss in a home setting [15]. The study is designed as a cluster-randomized trial in which all births attended by a peripheral health care cadre constitute a cluster.

## Methods

### Ethics Statement

The study protocol was approved by the Kintampo Health Research Center (KHRC) and the Ghana Health Service (GHS) Ethical Review Committees, the Institutional Review Board of the Johns Hopkins Bloomberg School of Public Health (#00002673), and the Research Ethics Committee of PATH (#HS547). The protocol is reviewed and reapproved annually.

### Study Design and Participants

This community-based, cluster-randomized trial was designed to determine if oxytocin (10 IU) by injection via Uniject by community health officers (CHOs) at home-based births in four rural districts in Ghana reduces the risk of PPH by 50% relative to CHO-attended deliveries where prophylaxis was not provided. Births attended by a CHO constitute a cluster. The random allocation was done at the cluster level (i.e., CHOs) rather than at the individual level (i.e., each delivering woman) so that each individual CHO was given a constant set of responsibilities, thereby simplifying the training and, most importantly, reducing the likelihood of inadvertent cross-over. We have previously published details on the design and implementation of this trial [16]. Briefly, the trial was conducted in four predominantly rural districts in Brong-Ahafo region. These are: Kintampo North, Kintampo South, Nkoranza North, and Nkoranza South. The maternal mortality ratio in that general area was 363 per 100,000 live births in 2008 [17]. The institutional birth rate in the study districts before enrollment commenced was 64%. National data from the Demographic and Health Surveys suggest that there is negligible use of skilled birth attendants at home births in Ghana [18]. Health facility delivery is free in Ghana. CHOs are employees of the GHS, trained for two years to provide outreach health services to the rural population, including childhood immunization, antenatal and postnatal care, and family planning. CHOs do not have midwifery skills. The majority of CHOs in this trial were recent CHO training program graduates.

Initially, we identified the catchment areas of 52 CHOs within study districts. KHRC field workers were responsible for the identification of all pregnant women. At seven or more months' gestation, field workers sought written initial informed consent for the trial. Women agreeing to participate, should they choose to deliver at home, were asked to contact the field worker after the onset of labor. There were no exclusion criteria. When contacted after onset of labor, the field worker telephoned the CHO and both travelled to the woman's household, where the written “final consent” process was administered. Enrolled women are those who provided final consent and whose delivery was observed by the CHO. The CHOs did not in any way manage the delivery; they (1) observed, (2) provided the intervention (according to random allocation), (3) measured the outcomes, and (4) provided early treatment for PPH and facilitated referral, when needed.

### Randomization and Masking

The 52 CHOs were randomly allocated equally to either the intervention (oxytocin) or the control group; this allocation was stratified by both district and distance (≤10 km or >10 km) to emergency obstetric care. The randomization sequence was determined using Stata (version 12). The random allocation sequence was generated by LCM, while enrollment of the clusters was done by SN and KHRC in collaboration with the GHS. Two additional CHO catchment areas were selected to replace non-compliant CHOs both of which were in the oxytocin group, following the single interim meeting of the Data Safety and Monitoring Board in December 2011. The new catchment areas were adjacent to those of the non-compliant CHOs with similar characteristics. The random allocation was not masked because the efficacy of oxytocin is not in doubt, use of a placebo was unacceptable to the GHS, and filling the Uniject device with saline has prohibitive testing requirements.

### Intervention

**Figure 1:**
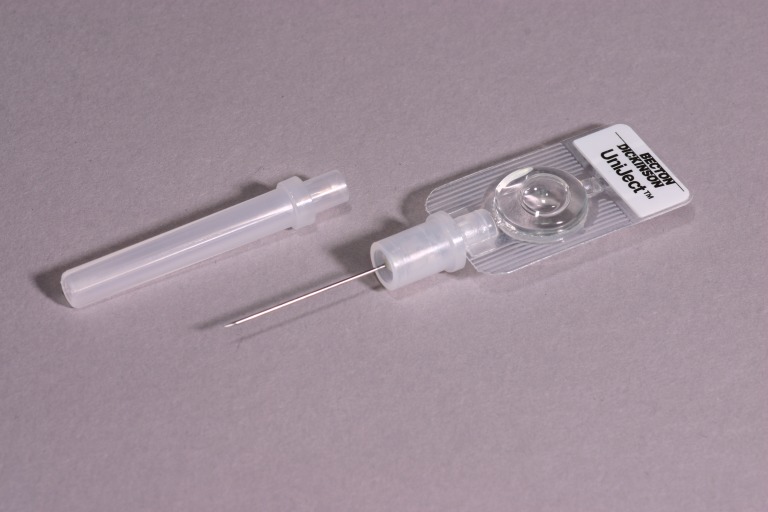
The oxytocin in Uniject device. *Source: PATH/Glenn Austin*.

The intervention was delivered by CHOs in the oxytocin group. The 28 CHOs allocated to this group administered an injection of oxytocin (10 IU) in the thigh one minute following delivery using an oxytocin in Uniject device, an auto-disposable syringe prefilled with oxytocin (10 IU) (Figure 1). The injection-ready format reduces wastage, simplifies logistics, and was designed for use both by lay health personnel who do not normally administer injections and in areas with limited health infrastructure. While the device has been used for observational studies [19]–[22], this is the first randomized trial using oxytocin in Uniject to evaluate impact on a health outcome. Supply of oxytocin in Uniject was manufactured by the Instituto Biológico Argentino (BIOL) and imported into Ghana for study purposes. A time/temperature indicator sticker was adhered to the foil envelope containing the device. This indicator is designed to change color with cumulative heat exposure to indicate when the drug should be discarded.

The 26 control group CHOs did not provide this prophylactic injection to women they observed. All other CHO activities (outcome measurement, data collection, and early treatment and referral when necessary, as described below) were identical across the control and oxytocin CHOs. All CHOs were required to successfully complete an allocation-specific, five-day competency-based assessment and were trained to palpate the uterus for a twin prior to administration of oxytocin. Oxytocin in Uniject supplies were kept refrigerated at KHRC and distributed approximately monthly in small quantities to CHOs, who stored them outside of a temperature-controlled cold chain at their CHO compound. CHOs requested additional supplies as needed from Field Supervisors.

### Response to Bleeding

CHOs in both groups were instructed to initiate emergency transport from KHRC if a woman was actively bleeding at 400 mL blood loss. Women who (1) lost more than 500 mL blood or (2) gushed blood and/or bled lime-sized blood clots received early treatment with oxytocin in Uniject (10 IU) prior to transport to one of four local hospitals. Emergency transport was also available from KHRC for other maternal and newborn complications.

### Outcome Measurement

CHOs in both groups measured postpartum blood loss by folding a BRASSS-V calibrated plastic drape under the woman's back prior to delivery. The drape was unfolded immediately after delivery so that the pouch could be used for blood collection. The drape has been validated and used in a number of previous PPH trials [15]. Women were asked to remain recumbent for one hour following delivery of the baby, or for two hours if active bleeding persisted. Fluids, urine, and feces were excluded from the blood-loss measure by sweeping them to the side and into a receptacle. CHOs in both groups were trained to monitor pulse, uterine tone, and vaginal bleeding every 15 minutes for the first two hours following delivery. Quantitative blood-loss measures and actions taken to respond to that blood loss were utilized to estimate PPH, the primary outcome, under three definitions:

1. PPH-1 is the traditional definition and includes blood loss ≥500 mL one hour following delivery, or after two hours if active bleeding persists at one hour. This traditional definition is problematic in two ways in this trial design: some women in both groups will be provided a treatment dose a) before completing one hour postpartum or b) prior to reaching 500 mL blood loss, potentially manipulating the total blood loss that might have occurred.
2. For this reason, PPH-2 includes PPH-1 plus any woman receiving early treatment for PPH regardless of cumulative blood loss. The denominator for PPH-1 and PPH-2 was all enrolled women with a quantitative blood-loss measure.
3. Furthermore, some women may be referred to a higher level of care for postpartum bleeding who are not included in either PPH-1 or PPH-2. PPH-3 includes any woman *without* a quantitative blood loss measure who was referred to higher care for postpartum bleeding, in addition to all women included in PPH-1 and PPH-2. The denominator for PPH-3 is all enrolled women.

Multiple definitions of PPH were therefore used to account for the impact on cumulative blood loss of early treatment for bleeding. In addition, we examined a variation of PPH-1, where the cutoff was extended to 1000 mL to represent severe PPH, given the common usage of this indicator.

Secondary outcomes assessed the safety and feasibility of home-based PPH prevention. Safety outcomes were analyzed for all enrolled women plus women who were referred to hospital before delivery of the baby. Women referred before delivery were included in the analysis of safety outcomes to account for the possibility of oxytocin administration by the CHO before delivery of the baby. Use of oxytocin before delivery of the baby was the key secondary outcome regarding safety. Others included maternal death, uterine rupture, stillbirth, early death of newborns (one to three days after birth), difficulty in infant's breathing at birth, need for newborn resuscitation, and needle stick injury. Fetal and infant outcomes were included as safety indicators out of concern regarding inappropriate use of oxytocin before delivery of the baby. Only basic indicators of feasibility were examined here, including the percentage of women who call for a CHO during labor, the percentage of deliveries for which the CHO arrived prior to delivery, mean duration of time spent (a) traveling to the household and (b) at the household, mean number of household visits per CHO, and mean response rate per CHO.

### Data Collection

Data were collected at three time points: at initial enrollment, during labor/delivery and immediate postpartum period, and two to three days after delivery. Field workers conducted interviews with women at the time of initial enrollment, which included socio-demographic information, pregnancy history, and use of health care. During the observation of women before and after birth, CHOs documented birth outcomes and the timing and completion of events and procedures using a structured questionnaire. All questionnaires were translated and back-translated from English to Bono/Twi and were pretested during a one month preparation phase preceding commencement of enrollment that permitted implementation of all field procedures except the intervention. Clocks with timers were distributed to CHOs to document timing of birth, injections, delivery of the placenta, and blood-loss measurements. Two to three days following delivery, field workers conducted separate follow-up interviews with all enrolled women and their traditional birth attendants (if one was present at birth) at their respective homes. These interviews were conducted as an independent assessment of the care provided to avoid relying on CHOs' documentation of their own behavior.

### Sample Size

Given existing data on the crude birth rate (27/1,000), an originally planned enrollment period of nine months, a mean population size per CHO catchment area (4,250), and an assumed proportion of deliveries at home and reachable by CHOs (28%), we estimated a mean cluster size of 24. Without information on PPH incidence, we estimated 10% based on a community-based trial in India [10], and conservatively set a coefficient of variation (k) = 0.35. Assuming a within-cluster loss to follow-up of 10%, we estimated that 26 CHOs per group were required for 80% power and 5% Type 1 two-sided error to detect a 50% reduction in PPH; over a 9-month period, we estimated that this sample design would yield 1,128 enrolled participants. In December 2011 (with 47% of the initial sample size achieved), our Data Safety and Monitoring Board met to examine trial progress. An external analyst assessed effectiveness of the intervention and confidentially presented results to the Board, which assessed them according to predetermined stopping guidelines (nominal alpha 0.00305, calculated using O'Brien-Fleming spending function). The trial was not stopped for efficacy or any other reason. The Board recommended increasing the sample size to account for 1) lower-than-expected enrollment per cluster, 2) lower-than-expected overall rate of PPH, and 3) imbalance in size of the oxytocin versus control clusters. In response, we extended data collection to 19 months (the maximum possible given available resources), estimating that this increase in the recruitment period would bring total sample size to 1,651. In a posthoc calculation, estimates of control-group incidence of PPH and coefficient of variation were updated at this time using data available at the interim analysis to re-estimate power to detect a 50% reduction in PPH-1 (67.6%) and PPH-2 (91.0%) given this increased sample size.

### Statistical Analysis

Analysis of primary and secondary outcomes was conducted following an intent-to-treat approach. Maternal, household, and socio-demographic variables; indicators of pregnancy history; and a range of variables related to care provided during pregnancy and prior to delivery were compared between the oxytocin and control clusters. Indicators of oxytocin and control-group implementation coverage and completeness were estimated for each group. Our analyses of the primary and secondary outcomes were conducted using individual-level data. The proportion of enrolled women with each of the binary definitions of PPH and of the secondary outcomes reflecting safety were estimated for each arm of the trial, and the risk ratio of each outcome in oxytocin versus control clusters was calculated using binomial regression with log link function. We did not adjust for multiple testing of the varying definitions of PPH. We additionally defined severe PPH as a quantitative blood-loss measure ≥1,000 mL and followed an identical analysis for between-group comparison. Mean blood loss was estimated for each group, and the difference between groups was estimated using linear regression. For all between-group comparisons using individual-level data, we estimated standard errors (and confidence intervals and p-values) by adjusting for clustered allocation using general estimating equations [23]. We additionally conducted a cluster-level analysis for the primary outcome, calculating the probability of PPH in each cluster and conducting a t-test with assumption of unequal variances to estimate the absolute difference in PPH between the groups. Stratified analyses included examination of PPH-1 and PPH-2 at 25%, 50%, 75%, and 100% of the cumulative sample size enrolled, and parity.

## Results

**Figure 2:**
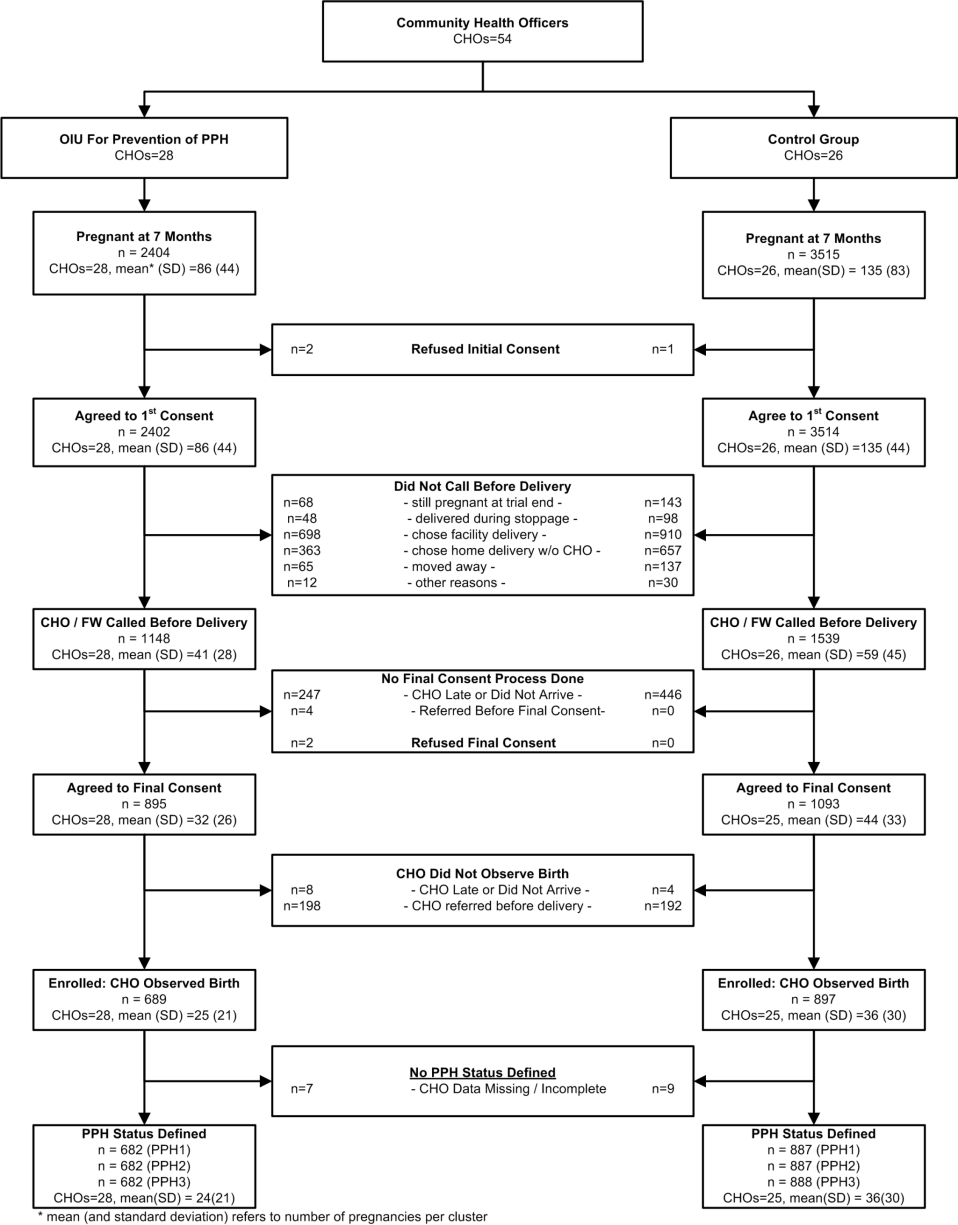
CONSORT flow diagram.

Enrollment was conducted between April 21, 2011 and November 30, 2012, as planned following the interim analysis. Figure 2 presents the trial profile. Community health officers were assigned to work in oxytocin (28 CHOs) or control (26 CHOs) areas. Field workers identified 5,919 pregnant women at seven months' gestation or more, nearly all (99.9%) of whom gave initial consent to participate in the study. The ratio of oxytocin to control women among the initially consented was 0.68 (2,402∶3514). Among the initially consented, 1,148 of 2,402 (47.8%) and 1,539 of 3,514 (43.8%) women in the oxytocin and control groups, respectively, requested CHO presence during labor. Among those who did not call for a CHO, 698 of 1,254 (55.7%) women in the oxytocin arm and 910 of 1,975 (46.1%) controls chose a health facility delivery. Among those who called for CHO presence at delivery, 689 of 1,148 (60.0%) women in the oxytocin group and 897 of 1,539 (58.3%) controls had their delivery observed by a CHO. The ratio of enrolled women in the oxytocin versus control arm was 0.77 (689∶897). The two predominant reasons for non-enrollment in both groups were: CHO did not arrive, and CHO referred the woman for facility-based care before delivery. Seven and nine enrolled women in the oxytocin and control arms, respectively, lacked a blood-loss measure.

The randomization process achieved balance between oxytocin and control clusters on a range of women's characteristics (Table 1) and on indicators of recruitment, enrollment, and measurement procedures (Table 2). Two CHO behaviors differed between oxytocin and control groups. Among CHOs called by women prior to birth, oxytocin CHOs referred women to hospital prior to delivery in 198 of 1,148 (17.3%) deliveries as compared to 192 of 1,539 (12.5%) deliveries referred by control CHOs. Likewise, in 58 of 689 (8.4%) enrolled oxytocin deliveries and 278 of 897 (31.1%) enrolled control deliveries, CHOs tied on the drape after delivery of the baby and before delivery of the placenta, as opposed to before delivery of the baby, per protocol. The mean duration between birth and unfolding the drape, which could potentially affect blood-loss measurement, confirms a delay of one minute on average by control CHOs relative to oxytocin CHOs (4.4 minutes [SD: 5.7] versus 3.4 minutes [SD: 5.4], respectively).

**Table 1:** Assessment of balance between the oxytocin and control groups achieved through the randomization process among enrolled women.

Enrollment Indicators/Characteristics	Oxytocin	Control
Woman's age (years) (m, SD)	27.5 (6.6)	26.9 (6.8)
*Woman's age group (n, %)*		
15–19	75 (10.9)	121 (13.5)
20–29	351 (50.9)	460 (51.3)
30–39	228 (33.1)	282 (31.4)
40–49	35 (5.1)	34 (3.8)
*Woman's education (n, %)*		
None	331 (48.0)	418 (46.6)
Some primary school	146 (21.2)	203 (22.6)
Middle/Continuation	195 (28.3)	264 (29.4)
Technical/Secondary/University	17 (2.5)	12 (1.3)
*Ethnic group (n, %)*		
Akan	211 (30.6)	309 (34.4)
Dagarti/Frafra/Kusasi	146 (21.2)	143 (15.9)
Gonja/Dagomba/Mamprusi	99 (14.4)	190 (21.2)
Konkomba/Basare	84 (12.2)	127 (14.2)
Mo	52 (7.5)	37 (4.1)
Other ethnicities*	97 (14.1)	91 (10.1)
*Marital status (n, %)*		
Married	446 (64.7)	503 (56.1)
Living together	168 (24.4)	331 (36.9)
Divorced/Separated/Widowed	14 (2.0)	10 (1.1)
Single/Unmarried	61 (8.9)	53 (5.9)
*Religion (n, %)*		
Catholic	153 (22.2)	164 (18.3)
Protestant	159 (23.1)	137 (15.3)
Pentecostal	169 (24.5)	263 (29.3)
Muslim	165 (23.9)	264 (29.4)
Traditional/Other	43 (6.2)	69 (7.7)
*Parity (live and stillbirth) (n, %)*		
No prior births	133 (19.3)	181 (20.2)
1	123 (17.9)	194 (21.6)
2 to 4	298 (43.3)	354 (39.5)
5 or more	135 (19.6)	168 (18.7)
Wealth score (0–10)** (m, SD)	3.6 (2.0)	3.8 (2.0)
*Previous stillbirth (n, %)*		
No prior pregnancy	115 (16.7)	161 (17.9)
None	549 (79.7)	695 (77.5)
Yes	25 (3.6)	41 (4.6)
*Previous early neonatal death (n, %)*		
No prior pregnancy	115 (16.7)	161 (17.9)
None	546 (79.2)	694 (77.4)
Yes	28 (4.1)	42 (4.7)
*Prior delivery location (n, %)*		
No prior pregnancy	115 (16.9)	161 (18.2)
Hospital/Health center	146 (21.5)	174 (19.7)
Home/Other	419 (61.6)	550 (62.1)
Missing	9	12
*Prior pregnancy antenatal care (ANC) (n, %)*		
No prior pregnancy	115 (16.9)	161 (18.2)
None	26 (3.8)	51 (5.8)
1 or more ANC visits	539 (79.3)	674 (76.1)
Missing	9	11
**Delivery indicators/characteristics**		
*Singleton/Multiples (n, %)*		
Singleton	669 (98.1)	879 (98.8)
Twins	13 (1.9)	11 (1.2)
Missing	7	7
*Reported size of baby* ***		
Smaller than normal	13 (1.9)	34 (3.9)
Normal	526 (78.7)	698 (79.4)
Larger than normal	129 (19.3)	147 (16.7)
Missing	21	18
*Birth attendant*		
Traditional birth attendant	647 (94.9)	862 (97.0)
Family member	27 (4.0)	21 (2.4)
Study participant herself	8 (1.2)	2 (0.2)
Other	0 (0.0)	4 (0.4)
Missing	7	8
*Receipt of traditional preparation to speed labor (n, %)*		
Yes	59 (8.7)	75 (8.4)
No	622 (91.3)	817 (91.6)
Missing	8	5
*Receipt of traditional preparation to stop postpartum bleeding (n, %)*		
Yes	2 (0.3)	3 (0.3)
No	667 (99.7)	866 (99.7)
Missing	20	28
*Receipt of pharmaceutical drug (from any provider) to speed labor (n, %)*		
Yes	0 (0.0)	0 (0.0)
No	680 (100.0)	887 (100.0)
Missing	9	10
*Receipt of pharmaceutical drug to stop bleeding postpartum (provided by someone other than the community health officer [CHO]) (n, %)*		
Yes	0 (0.0)	3 (0.4)
No	618 (100.0)	781 (99.6)
Missing	71	113
Report of duration of labor, in hours (m, SD)	3.9 (3.1)	3.8 (4.1)

* Includes: Bimoba/Chokosi, Fulani, Ga/Adangbe/Ewe, Sisala/Wala, Zambraba, Banda/Pantra, and other (non-specified).

** Score based on unweighted sum of 10 household assets (electricity, radio, cooker, refrigerator, television, sewing machine, cell phone, cement floor, cement walls, non-thatch roof).

*** Reported among singleton births only.

**Table 2:** Assessment of balance in recruitment, enrollment, measurement, coverage of the intervention, and early postpartum hemorrhage (PPH) treatment between groups.

Recruitment/Enrollment Indicator	Oxytocin	Control
**Among all women approached (n, %)**		
Refused initial consent	2 (0.1)	1 (0.0)
Agreed to initial consent	2,402 (99.9)	3,514 (100.0)
*Among all participating pregnancies*		
Called community health officer (CHO) or field worker (FW)	1,146 (47.7)	539 (43.8)
Did not call	1,256 (52.3)	1,975 (56.2)
*Among those that called CHO or FW*		
Enrolled	689 (60.3)	897 (58.3)
Not enrolled - CHO late or did not arrive	255 (22.3)	450 (29.2)
Not enrolled - referred before delivery	198 (17.3)	192 (12.5)
*Measurement Indicators*		
Status of placing the drape		
Before delivery	618 (90.0)	593 (66.3)
After delivery, before placenta	58 (8.4)	278 (31.1)
After delivery of placenta	4 (0.6)	12 (1.3)
Not tied/missing	7 (1.0)	11 (1.2)
Time between delivery and unfolding of the drape (minutes) (m, SD)	3.4 (5.4)	4.4 (5.7)
*Portion of first hour spent lying on back after drape applied*		
60 minutes	649 (96.4)	855 (98.2)
50–59 minutes	11 (1.6)	9 (1.0)
40–49 minutes	10 (1.5)	4 (0.5)
<40 minutes	3 (0.4)	3 (0.3)
*Quantitative blood measurement available (i.e., is PPH-1 defined)?*		
No	7 (1.0)	10 (1.1)
Yes	682 (99.0)	887 (98.9)
**Intervention Characteristics**		
*Received prophylactic injection*		
No	12 (1.7)	896 (99.9)
Yes	677 (98.3)	1 (0.1)
*Timing of prophylactic injection*		
Not received	12 (1.7)	896 (99.9)
After delivery, before placenta	667 (96.9)	1 (0.1)
After delivery, after placenta	9 (1.3)	0 (0.0)
*Location of prophylactic injection*		
Not received	12 (1.7)	896 (99.9)
Thigh	677 (98.3)	1 (0.1)
Time to prophylactic intervention (minutes) (m, SD)	1.79 (3.4)	21.00 (0.0)
*Early PPH Treatment*		
Among all women with PPH-3 (n, %)		
Received early treatment of oxytocin (10 IU)	24 (85.7)	90 (90.9)
Did not receive early PPH treatment	4 (14.3)	9 (9.1)

**Figure 3:**
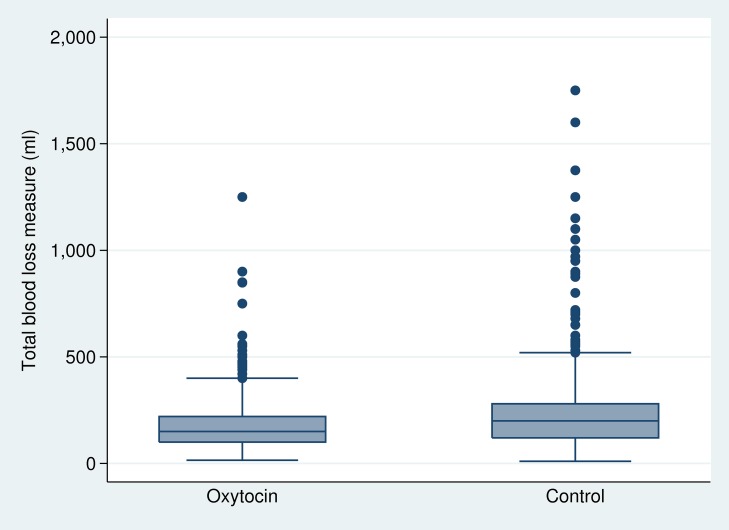
Median blood loss by study arm of the trial.

Primary outcome data were available for 682 women in the oxytocin group and 888 women in the control group (Table 3). The PPH-1 rate among women in the oxytocin group was 2.6% (18 of 682) versus 5.5% (49 of 887) among controls (RR: 0.491; 95% CI: 0.274–0.882). The PPH-2 rate among women in the oxytocin group was 3.8% (26 of 682) versus 10.8% (96 of 887) among controls (RR: 0.338; 95% CI: 0.181–0.630). The PPH-3 rate among women in the oxytocin group was 4.1% (28 of 682) versus 11.1% (99 of 888) among controls (RR: 0.361; 95% CI: 0.196–0.664). There were nine cases of severe PPH; all but one occurred in the control group (RR = 0.147; 95% CI: 0.013–1.70). Figure 3 presents a box plot illustrating the median, 25%, and 75% quartiles for blood loss by arm of the trial; median blood loss for those in the oxytocin group was 150 mL and among the controls it was 200 mL. Mean blood loss was 185.5 mL and 229.2 mL for oxytocin and control deliveries, respectively. Mean reduction in blood loss was 45.1 mL (95% CI: 17.7–72.6). When PPH-1 was examined in a cluster-level analysis, there was an absolute risk difference of 3.2% (95% CI: 0.3%–6.2%).

**Table 3:** Primary outcome measures, by group.

	Control			Oxytocin				
Outcome	N	Cases	Percentage (%)	N	Cases	Percentage (%)	Relative Risk (95% CI)	p-value
PPH-1	887	49	5.5	682	18	2.6	0.491 (0.274–0.882)	0.02
PPH-2	887	96	10.8	682	26	3.8	0.338 (0.181–0.630)	0.001
PPH-3	888	99	11.1	682	28	4.1	0.361 (0.196–0.664)	0.001
Severe PPH	887	8	0.9	682	1	0.1	1.000 (0.013–1.700)	0.12

Note: The intra-class correlation coefficients for PPH-1, PPH-2, and PPH-3 were 0.012, 0.051, and 0.050, respectively.

**Figure 4:**
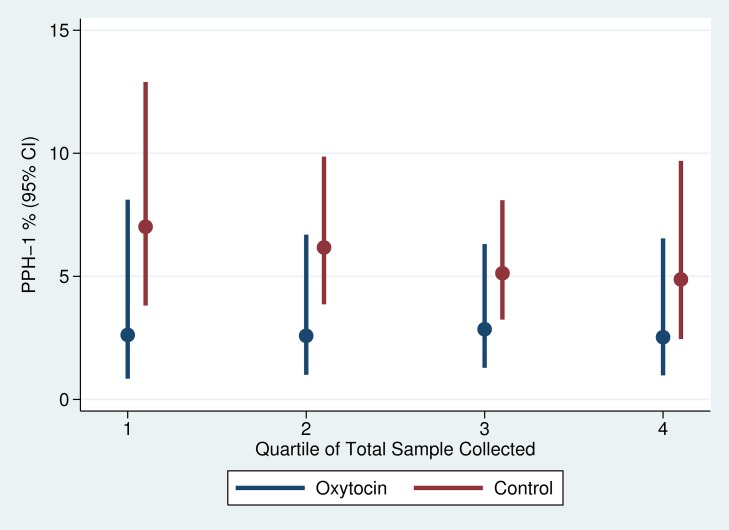
Trend in PPH-1 by study arm of trial.

**Figure 5:**
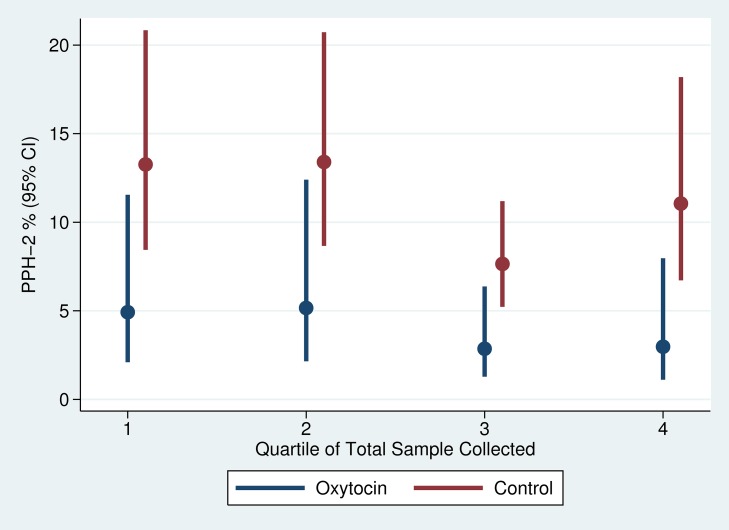
Trend in PPH-2 by study arm of trial.

There was slight variation in the quarter-specific estimates of PPH-1 and PPH-2, but no statistical evidence that these variations represented any secular trend in incidence in either the intervention or the control group (Figures 4 and 5). Similarly, the impact of prophylactic oxytocin on PPH rates did not vary across time period (p-value for time period interactions ranged from 0.51 to 0.89). The reductions in PPH-3 incidence were larger among primiparous women (RR: 0.231; 95% CI: 0.067–0.795) and women at parity five or higher (RR: 0.210; 95% CI: 0.054–0.821), compared to those with two to four births (RR: 0.461; 95% CI: 0.246–0.867); results were similar for PPH-1 and PPH-2, but in no case were the interaction terms statistically significant (Tables S1 and S2).

Stillbirth, early neonatal death (death prior to follow-up interview), difficulty breathing at birth, and need for infant resuscitation occurred at similar frequency across the groups (Table 4), and none of these showed a statistically significant relationship with receipt of prophylactic oxytocin. There were no cases of oxytocin use during labor, maternal death, uterine rupture, or needle stick injury. There were no major adverse events or unanticipated harmful events that required notification of the institutional review boards.

**Table 4:** Secondary safety outcome measures, by group.

	Control			Oxytocin				
Outcome	N	Cases	Percentage (%)	N	Cases	Percentage (%)	Relative Risk (95% CI)	p-value
Stillbirth	1,095	18	1.6	911	19	2.1	1.309 (0.851–2.016)	0.22
Early infant death (within 0–3 days)	1,077	7	0.6	892	6	0.7	1.000 (0.320–3.127)	1.00
Infant did not breathe at birth	891	48	5.4	684	45	6.6	1.129 (0.575–2.216)	0.72
Infant resuscitation*	891	26	2.9	684	11	1.6	0.632 (0.199–2.009)	0.44

* Defined as: clearing of infant airways, ventilation, cardiac massage.

Regarding intervention feasibility and success in service delivery, among initially consented women in the oxytocin and control groups 47.8% (1,148 of 2,402) and 43.8% (1,539 of 3,514), respectively, requested CHO presence at birth. The rate of health facility–based births in the CHO catchment areas remained stable over the 19-month period ranging from 34%–35% (n = 5919 deliveries). Among women requesting CHO presence at birth (in the oxytocin group n = 1,148 and in the control group n = 1,539), CHOs successfully arrived before delivery for 893 (77.8%) of oxytocin deliveries and 1,089 (70.8%) of control deliveries. Reasons for CHO non-compliance generally reflected scheduling or infrastructure issues (e.g., out of town, no cell phone coverage, phone battery dead, concurrent CHO outreach activities). On average, CHOs in the oxytocin and control groups responded to 1.7 and 2.2 calls per month, respectively. However, across CHOs the means per month in the oxytocin (n = 28 CHOs) and control groups (n = 26 CHOs) varied from 0.1–5.0 and 0–6.6, respectively. Likewise, the mean CHO response rate, defined per CHO as the percent of CHO household visits among all requests for service, was 69.9% (varying from 33%–100%) in the oxytocin group (n = 28 CHOs) and 69.7% (varying from 0%–100%) in the control group (n = 26 CHOs).

All cases of PPH should have received early treatment and been transported for higher level care; 24 of 28 (85.7%) of all PPH-3 cases in the oxytocin group and 90 of 99 (90.9%) in the control group received early treatment. However, only 35.7% (10 of 28) and 18.2% (18 of 99) women with PPH-3 in the oxytocin and control groups, respectively, were transported to hospital; reasons for non-compliance included bleeding stopped and/or the woman refused. There were no reported cases of CHOs using a Uniject device with a time/temperature indicator indicating “discard” in either group. In 1 and 2 oxytocin and control deliveries, respectively, the CHO did not provide an injection because the time/temperature indicator indicated “discard”.

## Discussion

This community-based, cluster-randomized trial of prophylactic oxytocin (10 IU) administered via Uniject by peripheral health care providers without midwifery skills showed statistically significant reductions of 51% in the risk of PPH for blood loss ≥500 mL and 66% for blood loss ≥500 mL or with gushing and large blood clots. Results for severe PPH (≥1,000 mL) were not statistically significant. All secondary outcomes suggested the intervention was safe.

The magnitude of protective effect for blood loss ≥500 mL in this trial is similar to results shown for community-based prophylactic misoprostol (600 mcg) versus placebo in rural India (47% reduction in PPH) [10] and to a meta-analysis of health facility–based prophylactic use of intramuscular and intravenous oxytocin versus no uterotonics in high-income countries (50% reduction in PPH defined as ≥500 mL blood loss; 95% CI: 0·43–0·59) [9]. PPH reduction in this study was greater than trial results shown for prophylactic misoprostol (600 mcg) versus placebo in rural Pakistan (24% reduction in PPH) and in Guinea Bissau (11% non-significant reduction in PPH) [11],[12]. Mean blood loss among both oxytocin and control women in this trial was lower than that reported in these community-based misoprostol trials. Our results show mean blood loss of 185.5 mL and 229.2 mL among oxytocin and control deliveries, respectively, as compared to results from India (214.3 mL versus 262.3 mL), Pakistan (337 mL versus 366 mL), and Guinea Bissau (443 mL versus 496 mL) [11]–[13]. Lower blood loss in this trial is expected, as early treatment of PPH with an additional injection of oxytocin (10 IU) in both our trial arms likely reduced ultimate blood loss among all PPH cases.

Several limitations of this study warrant discussion. The ratios of numbers of women in oxytocin and control arms of the trial range from 0.68 at the identification of eligible pregnancies to 0.77 at enrollment. Prior to randomization, CHO catchment areas were stratified by distance to emergency obstetric care, but not by population size, as these data were only available for Kintampo North and South. In these two districts, the ratio of population in the oxytocin versus control catchment areas is 0.88, explaining some, but not all, of the imbalance. As shown in Table 2, fertility between study arms is similar. There was no evidence that field workers in the oxytocin arm systematically missed identifying more pregnant women than in the control arm. However, if they did, it is unlikely that these women would have substantially different characteristics than the homogenous group that provided initial consent for the study (and which represented 99% of all identified pregnancies in both groups).

Two CHO behaviors differed by arm of the trial. First, control CHOs initiated blood-loss measurement (i.e., unfolded the drape) on average one minute later than oxytocin CHOs following delivery, possibly leading to an underestimate of blood loss in the control group. However, if delayed unfolding of the drape by control CHOs reduced blood loss measures, it would underestimate impact of oxytocin on outcomes. Second, CHOs in the oxytocin group referred women prior to delivery more frequently (17.3%) than control CHOs (12.5%), possibly indicating that the analyzable sample in the oxytocin group excluded a larger pool of women having a difficult delivery and thus potentially at higher risk of PPH. Data extracted from hospital records for referred women did not, however, indicate a higher risk (6 PPH cases among 198 referred women in the oxytocin group = 3.0%).

In an un-blinded study, differential measurement errors across arms of the trial are possible and thus, lack of blinding constitutes a study limitation. However, in this study the intervention was provided by CHOs who were not birth attendants. They were not responsible for managing the births nor were they responsible for the birth outcome, two issues which we believe would decrease the chances that they influenced the study outcome. The CHO's job was to respond to the call, to give (or not give) the injection and measure blood. They had no previous experience with birth, or visual blood loss estimation and possible associations with bad maternal outcomes.

This trial provides evidence that administration of intramuscular prophylactic oxytocin in Uniject by peripheral health care providers without midwifery skills can effectively decrease the risk of PPH at home births under research conditions. Furthermore, none of the secondary outcomes reflecting safety suggested that this intervention was unsafe. Across trial arms, service was successfully provided to three-quarters of all calls requesting CHO assistance. However, CHO compliance varied widely, and was likely due to CHOs working alone in their catchment area per trial protocol; that is, they were on call 24 hours per day for 19 months. In a scaled-up program, additional staffing or the ability to refer calls to a neighboring CHO would be required to increase and sustain CHO compliance. There is no evidence that the intervention decreased health facility–based births. However, 17% of pregnant women (n = 5919) chose to deliver at home without a CHO and research is needed to understand the barriers to reaching these women. Another concern was the frequent lack of compliance with free referral to hospital for PPH cases. Refusal of referrals was unexpected and underscores the importance of providing community-based early treatment to women reluctant to seek care outside the home.

Cost is an important component of intervention feasibility. Oxytocin in Uniject is currently commercially available only in a few Latin American countries and thus its eventual market-driven cost is unknown. The United Nations Commission on Life-Saving Commodities for Women and Children includes oxytocin among 13 medicines unavailable to women due to issues such as cost or supply because they are not subsidized by global bulk purchase agreements or advance market commitments. The Commission has developed specific recommendations to address these issues as well as to promote oxytocin in a Uniject-type device [24]. It is anticipated that oxytocin in Uniject will cost US$1.00 or less, and potentially substantially less once sustained demand is established. The oxytocin in Unijects used in this study, were purchased on a non-commercial basis at a cost of $1.40 per dose. A commercial price for the product, should sustainable demand emerge, could be lower as economies of scale play a significant role in the cost of producing pharmaceutical products.

These results also raise additional questions. For example, if oxytocin in Uniject is not an option, could providers entrusted to vaccinate children and provide other injections use a traditional syringe and ampoule for oxytocin administration? The skills required are the same as are issues regarding needle disposal. As a cost-saving measure, could a time/temperature indicator be placed on a flat of oxytocin ampoules (versus individual ampoules), since ampoules within a flat are generally exposed to the same environmental conditions? This issue is mentioned in the UN Commission on Life-Saving Commodities for Women and Children and will likely be determined by the willingness of pharmaceutical companies to allow it. Regarding the duration that oxytocin can remain out of the cold chain in hot climates, additional analyses from this trial are currently underway to assess the number of days that oxytocin can remain out of the cold chain under field conditions in central Ghana prior to indication by the time/temperature indicator that the device should be discarded. Such information will serve health care planners in determining the required resupply schedule and the feasibility of this schedule locally. Could a lay provider safely and effectively use oxytocin in Uniject? (A randomized trial assessing use of oxytocin in Uniject by traditional birth attendants to prevent PPH is underway in Senegal now; clinicaltrials.gov: NCT01713153.) Is the BRASSS-V calibrated plastic drape used here necessary or could a simplified drape that indicates only when treatment and referral are needed (versus a quantitative blood loss measure), or other PPH detection pads suffice? This choice will be determined by the objectives of local health planners and the ability to easily obtain or import supplies. In Ghana, local manufacture of the blood loss measurement drape is considered a priority for scaled-up use.

While the move towards use of skilled birth attendants is gathering global momentum, poverty and inequity—particularly in selected areas in countries—will remain issues for the foreseeable future. Prophylactic use of uterotonics to prevent PPH, the biggest killer of women during childbirth, is a key intervention and use of oxytocin as the drug of choice should be considered where its use is feasible. It is unhelpful to pose this issue as oxytocin versus misoprostol. The more appropriate question is: Where and under what circumstances can each of these proven effective drugs be used? Ultimately, decisions regarding the balance of advantages and disadvantages of using oxytocin or misoprostol for PPH prevention at home births will depend on local conditions, human resources, infrastructure, and national policies.

## Supporting Information

Table S1 **Effect of the intervention on PPH-1 and PPH-2, stratified by parity.**
(DOCX)

Table S2 **Effect modification: parity on the effect of the intervention on PPH-1, PPH-2, and PPH-3.**
(DOCX)

Text S1 **Ghana Oxytocin Initiative trial proposal.**
(DOC)

Text S2 **Ghana Oxytocin Initiative CONSORT checklist.**
(DOCX)

## Abbreviations

KHRC: Kintampo Health Research Center
PPH: postpartum hemorrhage
