# Supplementary material for: Effect on Postpartum Hemorrhage of Prophylactic Oxytocin (10 IU) by Injection by Community Health Officers in Ghana: A Community-Based, Cluster-Randomized Trial
Source: PLoS Med. 2013 Oct 1;10(10):e1001524. doi: 10.1371/journal.pmed.1001524 (PMC3794862; doi:10.1371/journal.pmed.1001524)
Supplement: Table S1 — Effect of the intervention on PPH-1 and PPH-2, stratified by parity. (DOCX) [file pmed.1001524.s001.docx]

Supplemental Table 1: Effect of the intervention on PPH-1 and PPH-2, stratified by parity

| Outcome | Cases | RR | 95% CI | p-value |
| --- | --- | --- | --- | --- |
| PPH-1 |  |  |  |  |
| Primiparous | 308 | 0.277 | 0.062-1.228 | 0.91 |
| 1-4 births | 961 | 0.723 | 0.343-1.522 | 0.39 |
| 5+ births | 300 | 0.146 | 0.028-0.771 | 0.02 |
| PPH-2 |  |  |  |  |
| Primiparous | 308 | 0.278 | 0.078-0.981 | 0.05 |
| 1-4 births | 961 | 0.437 | 0.230-0.829 | 0.01 |
| 5+ births | 300 | 0.120 | 0.018-0.812 | 0.03 |
