# Supplementary material for: Effect on Postpartum Hemorrhage of Prophylactic Oxytocin (10 IU) by Injection by Community Health Officers in Ghana: A Community-Based, Cluster-Randomized Trial
Source: PLoS Med. 2013 Oct 1;10(10):e1001524. doi: 10.1371/journal.pmed.1001524 (PMC3794862; doi:10.1371/journal.pmed.1001524)
Supplement: Table S2 — Effect modification: parity on the effect of the intervention on PPH-1, PPH-2, and PPH-3. (DOCX) [file pmed.1001524.s002.docx]

Supplemental Table 2 Effect modification: parity on the effect of the intervention on PPH-1, PPH-2 and PPH-3

| Outcome | N | RR | 95% CI | p-value |
| --- | --- | --- | --- | --- |
| PPH-1 | 1569 |  |  |  |
| Intervention group |  | 0.329 | 0.074-1.454 | 0.14 |
| Primiparous |  | 1.042 | 0.506-2.146 | 0.91 |
| 5+ births |  | 1.302 | 0.558-3.037 | 0.54 |
| Intervention * primiparous |  | 2.068 | 0.429-9.965 | 0.36 |
| Intervention * 5+ births |  | 0.345 | 0.029-4.102 | 0.40 |
| Constant |  | 0.052 | 0.027-0.100 | 0.000 |
| PPH-2 | 1569 |  |  |  |
| Intervention group |  | 0.264 | 0.078-0.894 | 0.03 |
| Primiparous |  | 1.104 | 0.687-1.772 | 0.68 |
| 5+ births |  | 1.115 | 0.625-1.992 | 0.71 |
| Intervention * primiparous |  | 1.537 | 0.470-5.024 | 0.48 |
| Intervention * 5+ births |  | 0.629 | 0.123-3.221 | 0.58 |
| Constant |  | 0.110 | 0.067-0.179 | 0.008 |
| PPH-3 | 1570 |  |  |  |
| Intervention group |  | 0.244 | 0.075-0.787 | 0.02 |
| Primiparous |  | 0.947 | 0.610-1.470 | 0.81 |
| 5+ births |  | 0.961 | 0.554-1.668 | 0.89 |
| Intervention * primiparous |  | 1.775 | 0.573-5.494 | 0.32 |
| Intervention * 5+ births |  | 0.952 | 0.220-4.115 | 0.95 |
| Constant |  | 0.128 | 0.082-0.202 | 0.000 |
