# Supplementary material for: Effect on Postpartum Hemorrhage of Prophylactic Oxytocin (10 IU) by Injection by Community Health Officers in Ghana: A Community-Based, Cluster-Randomized Trial
Source: PLoS Med. 2013 Oct 1;10(10):e1001524. doi: 10.1371/journal.pmed.1001524 (PMC3794862; doi:10.1371/journal.pmed.1001524)
Supplement: Text S1 — Ghana Oxytocin Initiative trial proposal. (DOC) [file pmed.1001524.s003.doc]

**JHSPH Institutional Review Board**

**RESEARCH PLAN**

**Co-PIs:** Cynthia Stanton, PhD, Department of Population, Family, and Reproductive Health

Sam Newton, MD, PhD, Kintampo Health Research Center (Kintampo, Ghana)

**Co-Investigators:** Luke Mullany, PhD, Department of International Health

Sadaf Khan, MBBS, MPH, DrPH, PATH, Seattle, Washington

Patience Cofie, MSc, PATH (Accra, Ghana)

**Study Title:** The Oxytocin Initiative: Determining the effect of prophylactic administration of oxytocin in Uniject™ on postpartum hemorrhage at the community level in four districts in Ghana

**IRB No.:** IRB0002673

**PI Version Number/Date:** Version 9, February 27, 2012

1. **Aims/objectives/research question/hypotheses:**

**Primary objective:**

- 1. To determine if intramuscular administration of 10 IU of oxytocin in Uniject™ during the third stage of labor by a Community Health Officer (CHO) will reduce the risk of postpartum hemorrhage, defined as blood loss of 500ml or more within two hours after delivery, by 50% relative to deliveries attended by the same type of provider who does not provide the intervention. Postpartum hemorrhage is examined under three sequential definitions: 1) blood loss >500ml (BL); 2) treatment for bleeding (TX) and/or BL; 3) hospital referral for bleeding and/or TX and/or BL.

**Secondary objectives:**

- 1. To document the frequency of inappropriate use of oxytocin (administration prior to the delivery of the baby) by CHOs.
  2. To document the occurrence of adverse maternal and fetal/newborn outcomes which are associated with inappropriate and appropriate use of oxytocin by CHOs.
  3. To document logistical issues pertaining to expanded coverage of oxytocin for postpartum hemorrhage prevention, i.e., the arrival of CHOs in time to intervene, adequate storage, appropriate disposal of the Uniject™ device and changes over time in CHO practice.
  4. To assess the reliability of women’s self-report on medical and traditional care received during labor and delivery.

1. **Background and rationale:**

Postpartum hemorrhage is a leading cause of maternal deaths worldwide and yet is one of the few complications with an effective *preventive* intervention. Active management of the third stage of labor (AMSTL), defined as intramuscular administration of 10 IU of oxytocin, controlled cord traction and fundal massage, substantially reduces the risk of postpartum hemorrhage. A meta-analysis from four facility-based clinical trials showed a 62% reduction in the risk of postpartum hemorrhage (blood loss 500+ml).[1] A multi-center randomized controlled trial of AMTSL in hospitals in Argentina and Uruguay showed a 55% decrease in the median PPH rate ratios across hospitals in the intervention compared to the control arm. [2] The International Federation of Gynecologists and Obstetricians and the International Confederation of Midwives both recommend AMTSL provided by a skilled birth attendant for all singleton births.[3]

Oxytocin, the uterotonic drug of choice for AMTSL according to the World Health Organization (WHO), FIGO and the ICM, is a naturally occurring hormone in the human body that is stored in the pituitary gland. When released in a woman just after the birth of a baby, this hormone stimulates both milk production and contraction of the uterus to slow and stop uterine bleeding. Synthetic oxytocin is the form used in preventing and treating PPH. It is administered either intramuscularly or intravenously. Oxytocin has the most rapid onset of action of any of the medicines available for PPH. When given intramuscularly, it is effective two to three minutes after injection, and if given in the postpartum it has minimal side effects and can be used with all women. Side effects with oxytocin are not common when oxytocin is given after the baby is born. Serious side effects that are extremely rare, include: an [allergic reaction](http://www.emedicinehealth.com/script/main/art.asp?ArticleKey=59357) (shortness of breath; closing of the throat; hives; swelling of the lips, face, or tongue; [rash](http://www.emedicinehealth.com/script/main/art.asp?ArticleKey=101047); or [fainting](http://www.emedicinehealth.com/script/main/art.asp?ArticleKey=58678)); difficulty urinating; [chest pain](http://www.emedicinehealth.com/script/main/art.asp?ArticleKey=58673) or irregular heart beat; difficulty breathing; confusion; sudden weight gain or excessive swelling; severe [headache](http://www.emedicinehealth.com/script/main/art.asp?ArticleKey=59405); excessive [vaginal bleeding](http://www.emedicinehealth.com/script/main/art.asp?ArticleKey=58758); or seizures. Oxytocin reduces the length of the third stage of labor. In summary, synthetic oxytocin is one of the most important drugs available for obstetric purposes. In addition to its use in preventing and treating PPH, it is also administered intravenously for labor induction and augmentation. Attesting to its safety, oxytocin is on the essential drug list for obstetric purposes in virtually every country in the world.

AMTSL is not recommended for use by non-professional birth attendants due to concern regarding the risk of uterine inversion from controlled cord traction. In the absence of a skilled birth attendant who can provide all of the components of AMTSL, the World Health Organization (WHO) recommends that oxytocin or misoprostol should be given by a health worker trained in its use to prevent postpartum hemorrhage, and states further that oxytocin is preferred to misoprostol where its use is feasible. [4] Buekens reinforces this stance by emphasizing “that both drugs have their place in the prevention and treatment of PPH. However, oxytocin is the drug of choice and every effort should be made to make it widely accessible, including at the community level”. [5]Research is thus needed to assess the effectiveness, safety and feasibility of adapting use of oxytocin for births in peripheral facilities and at home.

Ghana is an excellent context in which to assess the effectiveness and safety of using home-based health care providers to administer injectable oxytocin in Uniject™ for the prevention of postpartum hemorrhage. Pregnancy-related mortality is high. A special Demographic and Health Survey on maternal health conducted in 2007 estimated the pregnancy-related mortality ratio to be 416 deaths per 100,000 live. [6] This same survey attributed 24% of maternal deaths to obstetric hemorrhage. Approximately 45 percent of births occur at home, and approximately 30 percent are assisted by a lay birth attendant, a proportion which has not changed since the mid 1980’s. [6]

Furthermore, in Ghana there are Community Health Officers (CHOs) who are trained as community nurses, and who already provide services such as immunizations, family planning, antenatal/postnatal care, treatment of minor ailments and health education. Approximately four percent of CHOs have been trained to manage deliveries, either based on training they received prior to becoming a CHO or as part of their CHO training. It is likely that more CHOs will receive midwifery training in the future. CHOs are supported by community volunteers who assist with community mobilization, the maintenance of community registers and other essential activities. CHOs are becoming more available throughout the country, and provide an excellent option for the provision of trained assistance at home births. Senior high school graduates are usually enrolled and offered a two-year theoretical and practical training on preventive and curative methods. They are then attached to and trained at a functioning Community Health Compound for at least six months before they are fully registered by the Nurses and Midwives council, and then posted to take charge of a new Community Health Program (CHPS) zone.

There are three key concerns regarding oxytocin use in peripheral settings; 1) it requires refrigeration, 2) it requires injection safety practices, and 3) it may be administered prior to delivery of the baby to induce and/or augment labor. The use of the pre-filled, auto-disable oxytocin (10 IU) in the Uniject device with a time-temperature indicator (TTI) alleviates many of the barriers associated with the first and second concerns.[7] It has been used extensively and safely by minimally trained personnel in vaccination campaigns in low resource settings. Oxytocin 10 IU in Uniject has been used in studies with doctors and midwives at the facility-level in Angola, and with lower level health professionals in Indonesia, Mali and Viet Nam. The TTI is a sticker adhered to the foil pouch containing each Uniject device. It changes color with cumulative exposure to temperature and indicates when the dose of oxytocin in Uniject has been exposed to excessive heat.[8] [9] Studies have shown that oxytocin contains more than 85% chemically active drug when stored below 30 degree Celsius for one year.[10] Thus, with access to some refrigeration or even coolers, and an appropriate distribution system, it is now feasible to consider using oxytocin in Uniject in peripheral settings, including where frequent re-supply is not feasible.

Use of any intramuscular uterotonic drug before the birth of the infant is regarded as dangerous because it is not possible to adjust the dosage if it causes hyper-stimulation and cannot be adapted to the level of uterine activity, as is possible with the monitored administration of intravenous oxytocin in health facilities.[11] Hyper-stimulation of the uterus can lead to uterine rupture, fetal asphyxia or fetal demise.[12] [13] These consequences are the basis for the third concern, use of oxytocin prior to delivery of the baby. A recent literature review shows the unsupervised use of uterotonic drugs at home births in South Asia is commonplace, but this has rarely been documented in sub-Saharan Africa [14]. An informal poll of Ghanaian members of the Technical Advisory Group for this study, staff at the Ghana Health Service and health care providers working in Ghana reported that such use was unknown in Ghana. Too few data were identified in the literature review regarding use of uterotonics for labor induction or augmentation at peripheral facilities in sub-Saharan Africa to reach a conclusion. Thus, although the introduction of pharmaceutical uterotonic use at the home could potentially lead to inappropriate use, in Ghana the evidence that is available suggests that this behavior is not already well established. Hence, concerns regarding the safety of providing increased access to pharmaceutical uterotonic drugs are diminished as this is a setting where inappropriate use appears to be unknown. By selecting a uterotonic-naïve setting, one has greater ability to introduce safe home-use of oxytocin via the education components of the intervention. Lastly, results from this proposed study are likely to be generalizable as these characteristics are common across many sub-Saharan African countries.

There are some unpublished studies, as well as anecdotal information from midwives in Ghana, that women turn to traditional preparations (herbs, roots, etc.) to hasten labor. It is not known whether these traditional preparations actually contain uterotonic/pharmacological properties. Biochemical assessment of traditional preparations used at birth is difficult as the chemical qualities of the plants used will vary with rainfall and other environmental conditions and dosage is far from standardized. No literature was identified which suggested possible interactions between oxytocin and such traditional preparations.

***Rationale for the design:***

This trial is designed to assess the effectiveness of a mode of service delivery. That is, can the evidence-based practice of using oxytocin for PPH prevention be effectively delivered by a low level health care cadre at home births? We aim to assess whether this mode of service delivery can a) achieve a health benefit *and* b) be delivered safely *and* c) whether this mode of delivery is feasible from a practical and logistical standpoint. PPH is the primary outcome of this study for two reasons: 1) there is general consensus regarding the outcome (>500mls of blood loss constitutes PPH) and 2) validated tools exist for the measurement of blood loss in a home setting. Although the safety of this intervention is as important as its health benefit, there is no consensus on what is an unacceptable level of mistimed use of oxytocin (in a setting without fetal monitors). Thus, the study is designed with safety as a secondary objective.

The decision to design the study as a randomized trial was based on 1) a stance of equipoise regarding the ability of CHOs with local infrastructure to effectively deliver this intervention and 2) the need for a rigorous study assessing the safety and effectiveness of expanding oxytocin to the periphery. Oxytocin 10 IU in the Uniject device has existed for more than a decade, and it is very clear that Ministries of Health, non-governmental organizations involved in health care provision and international donors are not going to take this intervention forward without data from a rigorously designed study. To assure international and country-specific recognition of and input into this study, an International Technical Advisory Group consisting of 18 members with varied expertise in obstetrics, trial design, pharmaceutical supply, postpartum hemorrhage and maternal health programming was established, as well as a Ghana-specific Technical Advisory Group. Both groups meet annually. The International Technical Advisory Group has met twice thus far and the Ghana-specific group has met once. The International group has assisted greatly in the design of this trial, as well as the sister trial being conducted in Karnataka, India. In the future, both groups will also provide guidance on appropriate dissemination and policy implications of the results of this trial.

1. **Participants**:
2. ***Study participants*** will consist of pregnant women delivering at home in the presence of a family member and/or traditional birth attendant AND a CHO in demographic surveillance (DSS) areas of the following districts: Kintampo North and South and Nkoranza North and South. District maps are included in Appendices A and B. The Kintampo Health Research Center (KHRC) is one of the three well established field research sites of the Ghana Health Service and has been functioning since 1994. In these districts females constituted approximately 50% of the population with males making up 49%, which is consistent with the national average. Kintampo North and South and Nkoranza North and South districts are predominantly rural with approximately 20% the population residing in urban areas. Subsistence farming is the main activity as most members of the population are uneducated. Less than 10% of the women have completed any training basic training. Ninety-two percent of births to women in these districts received at least one antenatal care visit (and nationally, 77% of births to women received four or more antenatal care visits). Tetanus toxoid immunization coverage in these districts is at 83 percent as of 2009.
3. **Inclusion/exclusion criteria:** Inclusion criteria include: For initial enrollment, all pregnant women at or around seven months gestation who consent to participate in the study should they deliver at home. To reaffirm consent and for final enrollment, all pregnant women who deliver at home in the presence of a CHO and who reaffirm consent to participate in the study. Pregnant women at the time of delivery will be considered emancipated adults, regardless of age. There are no specific exclusion criteria. The percent of women who request CHO presence at birth but for whom a blood loss measurement is not possible due to the late arrival of the CHO, for example, will be reported among the indicators of the feasibility of this intervention.

**Original sample size:** The original sample size for this study was 1,128 pregnant women at the time of birth; Table 1 and the text below summarize those sample size and power calculations. With this amendment of 3/2012, the sample size has been re-estimated. The details and rationale for re-estimating sample size is discussed below under the sub-heading: Re-estimated Sample Size.

The sample size calculation assumes a postpartum hemorrhage rate of 10 percent, based on a community-based study in rural India that found an acute postpartum hemorrhage rate of 12% amongst its control group. [15] The efficacy of the intervention in the oxytocin/CHO clusters is assumed to be 50%, somewhat less than has been shown in hospital-based studies. Loss to follow up is estimated at 10% and the study is designed to be completed in nine months. It is estimated that 52 CHOs is required to achieve 1128 deliveries within nine months. Two additional CHOs will be randomized (for a total of 54) to allow for absences from their post. It is anticipated that each CHO will attend approximately 2.4 deliveries per month (125 deliveries per month across 52 CHOs over nine months). Although the CHOs have approximate catchment areas for which they are responsible, all home births attended by a CHO are potentially eligible for the study, regardless of the geographic location of the birth.

Table 1. Sample size assumption and power calculation

| ***ASSUMPTIONS*** | | |  |
| --- | --- | --- | --- |
| **Inferential Error Assumptions** | |  |  |
|  | Type 1 |  | ***0.05*** |
|  | Power (1-Type II) |  | ***0.8*** |
| **Demographic Assumptions** | |  |  |
|  |  |  |  |
|  | Crude Birth Rate |  | ***27*** |
|  | - Proportion of Births observed by CHOs |  | ***28%*** |
|  | - "CHO Observed Birth Rate" |  | ***8*** |
|  | A-priori length of comparative phase |  | ***0.75*** |
|  | Mean population size covered by a CHO |  | ***4250*** |
|  | Mean number of CHO-observed births per year |  | ***32*** |
|  | Mean Cluster Size for Design Effect Calculation |  | ***24*** |
| **Intervention Efficacy Assumptions** | |  |  |
|  | Baseline Rate of PPH (i.e. Rate in Control Clusters) |  | **10.0%** |
|  | Efficacy of Intervention in Oxytocin in Uniject Clusters |  | ***50.0%*** |
| **Study Design and Follow Up Assumptions** | |  |  |
|  | Between cluster coefficient of variation ("k") |  | ***0.35*** |
|  | Loss to Follow Up |  | ***10%*** |
| ***SAMPLE SIZE ESTIMATIONS*** | | | |
| **Resulting Risk of PPH given assumptions** | |  |  |
|  | A. Risk among Control Clusters |  | ***0.10*** |
|  | B. Risk among Oxytocin in Uniject Group |  | ***0.05*** |
| **Primary Research Question:** A vs B | |  | ***A vs B*** |
| **Sample Size and Duration Estimation** | |  |  |
|  | Sample Size Required per Group (Clusters) |  | ***24*** |
|  | Sample Size Required per Group - after LFU |  | ***26*** |
|  | Groups to compare |  | ***2*** |
|  | Total Clusters (CHOs) Needed |  | ***52*** |
|  | Years Needed |  | ***0.75*** |
|  | Total Deliveries in Time Period |  | ***1,128*** |
|  | Total Deliveries if "k" was zero |  | ***911*** |
|  | Estimated Design Effect |  | ***1.2381*** |

**Re-estimated sample size**: Following the interim analysis and discussion at the DSMB meeting (12/15/2012), the DSMB recommended that sample size be re-estimated and data collection be extended to address power concerns resulting from an imbalance in enrollment between the intervention and control arms of the trial. (As of 10/2011, the ratio of intervention versus control CHO deliveries was 0.69). We examined what impact extending our study (up to various new end dates throughout 2012) would have on our power to detect 50% reductions in PPH1 and PPH2, given updated (i.e. interim analysis) information about parameters originally estimated before the study. We did not do a conditional power analysis; rather, we simply re-estimated parameters including control group incidence, the observed skew in enrollment rates between the groups, and new information on variation in size of the clusters. Our donor approved the use of resources to continue enrollment up to November 30^th^, 2012. We estimate that by November 30^th^, 2012, we will have enrolled approximately 1679 women. As this approximation is subject to variation in true pregnancy/birth rates within the population, ***we are requesting that the new approved sample size be set at 1679 + 10% = 1850 women***. Our power curves (see below) indicate that by November 2012, we will have between 40% and 60% power to detect 50% reductions in PPH1, and between 75% and 88% power to detect 50% reduction in PPH2, depending on actual intraclass correlation coefficient.

**Figure 1: Power to detect differences between groups in incidence of PPH1, by sample size available (reflected by date in 2012)**

**Figure 2: Power to detect differences between groups in incidence of PPH2, by estimated sample size available (reflected by date in 2012)**

1. **Identifiers:** There will be five types of individual level data collection forms, all of which will require names, addresses and study-related identifiers. Identifiers are required to permit follow up at home. List of questions for each of the following questionnaires are included in separate files as part of this application:
   - **Questionnaire #1: Potential participant identification form:** a short form completed by the field worker when a household with a pregnant woman is identified. Information on this form is restricted to identifiers to allow follow up later in pregnancy.
   - **Questionnaire #2: Background Questionnaire at enrollment in late pregnancy:** a short questionnaire administered by the field worker for all women at or around seven months gestation consenting to participate in the study. The questionnaire covers background characteristics of the participant.
   - **Questionnaire #3: CHO Questionnaire at time of delivery**: a structured questionnaire which will document identifiers for the woman and the provider, social/demographic indicators, traditional and medical health care received from the onset of labor through 1 hour postpartum, blood loss within one hour of delivery of the baby (or two hours if bleeding is on-going at one hour), fetal and maternal outcomes including descriptors of emergency referral. This questionnaire will be completed by the CHO during and after delivery.
   - **Questionnaire #4: Follow up questionnaire to be administered to the woman/family within 48-72 hours of delivery:** a structured questionnaire documenting identifiers, health care received from onset of labor through one hour postpartum (or through two hours if bleeding is on-going at one hour), fetal and maternal outcomes up through time of interview. This questionnaire will be administered by the field worker. In the event of a maternal death, a Follow Up questionnaire has been adapted for use with a family member. In addition, the WHO standard verbal autopsy questionnaires will be used to determine cause of death for women, stillbirths and deaths to live borns in the first week of life.
   - **Questionnaire #5: Follow up questionnaire for the non-family member, lay birth attendant within 48-72 hours of delivery:** a structured questionnaire documenting identifiers, health care received from the onset of labor through one hour postpartum (or through two hours if bleeding was on-going at one hour), fetal and maternal outcomes up through time of interview. This questionnaire will be administered by the field worker.
2. **Study procedures:**
3. **General study design and methods:**

This study was originally designed as a cluster-randomized, community-based trial, conditionally followed by a three month post-comparative universal distribution phase if the intervention is shown to be effective and if members of the Data Safety and Monitoring Board deem this to be appropriate. At the suggestion of the DSMB following the Interim Analysis on 12/15/2012, the conditional Universal Distribution Phase has been eliminated from the protocol (see Section 4 below), as the resources allocated for that Phase must be used to accumulate a larger sample size to improve power to detect any true differences in PPH between the groups. All home-based births attended by a CHO will constitute a cluster for the purpose of this trial. CHOs will be randomized into one of two study arms which will determine the treatment that CHO-assisted home births receive. Fifty-four CHOs will be randomly selected into the intervention or control groups using a computerized selection procedure assuming equal numbers in both groups. Given that the interventions in the two study arms are visible to all present, blinding is not an option for this study. Although the CHOs have approximate catchment areas for which they are responsible, all home births attended by a CHO are potentially eligible for the study, regardless of the geographic location of the birth. The treatment within each arm is described below:

1. Prevention and early treatment arm: One injection of 10 IU of oxytocin administered via Uniject™ within one minute after delivery of the baby (after first asking the woman if she is pregnant with twins which will be done shortly after arrival at the household and after ascertaining via abdominal palpation that there is no second baby. Abdominal palapation will be done for 100% of women prior to receiving oxytocin injection for prevention or treatment of postpartum hemorrhage). If the woman is actively bleeding (i.e. is experiencing a steady flow) when she has lost 400 mls of blood, referral must be initiated via phone call for emergency transport. To identify active bleeding, the CHO will monitor pulse, uterine tone and vaginal bleeding every 15 minutes for the first two hours. It will be verified that all CHOs have cell phones and units will be provided to them for their participation in this study. KHRC will have a vehicle and driver specifically available for this purpose who will immediately respond to the call. In the event of postpartum hemorrhage (defined as 500mls blood loss), early treatment with one additional injection of 10 IU of oxytocin and fundal massage will be provided by the CHO. In the event of gushing blood, uterus is neither hard nor round and/or clots the size of a lime, following delivery of the baby, CHOs will be trained to administer one injection of 10 IU of oxytocin and fundal massage and will initiate emergency referral, without waiting to verify that 500ml blood loss has been reached. Palpation for a second twin will be done prior to all injections of oxytocin. Emergency referral and transport will be available to women and newborns in both arms of the study for any type of complication. CHOs will be trained to recognize the cause for such referrals before the beginning of the study. CHOs may initiate emergency referral and transport at any time during the intrapartum and immediate postpartum periods per their judgment, based on the training they will receive as part of the Oxytocin Initiative. In the event of an emergency transfer, CHOs will accompany the woman to the hospital and remain there until the woman is admitted to the hospital. For each case of referral, supervisors will verify that referral procedures were followed and documented and any deviations from the protocol will be discussed with the CHO during weekly meetings with the supervisor. Seven days after admission, the CHO supervisor will be responsible for following up with hospital staff in order to document outcomes following the woman or newborn’s discharge from the hospital.
2. Early treatment only arm: No use of a uterotonic drug before or after delivery of the baby, except in cases of postpartum hemorrhage, in which case the CHO will provide early treatment (after first asking the woman if she is pregnant with twins which will be done shortly after arrival at the household and after ascertaining via abdominal palpation that there is no second baby) and emergency referral/transport as described for the first study arm. Again, emergency referral and transport for any complication will be available to all women and newborns at any point during the intrapartum and immediate postpartum periods.

Once 50% of the targeted sample size is achieved, a preliminary analysis of results will be conducted to assess effectiveness of the intervention. Effectiveness will defined as a 50% reduction, with a required significance level of 0.00305 in the risk of postpartum hemorrhage among those having received prophylactic oxytocin via Uniject at CHO-assisted births compared to CHO-assisted births without prophylactic use of oxytocin. Results will be summarized in a report submitted to the Data Safety and Monitoring Board within three months after having achieved 50% of the targeted sample size.

1. **Study procedures, sequence and timing:**

*Procurement, import, and distribution of oxytocin in Uniject:* Adequate quantities of the oxytocin in Uniject™ device for this study will be produced by BIOL pharmaceuticals of Argentina. Each Uniject™ device will include a time/temperature indicator on the outside of the foil pouch. The product will be imported as clinical study supplies into Ghana, and a clinical sample import permit will be obtained from the Ghana Food and Drug Board. Medicines requiring refrigeration are cleared of customs within 24 hours in Ghana. KHRC staff will be responsible for obtaining the oxytocin/Uniject devices from customs and transporting them to Kintampo in an air conditioned vehicle. The devices will be refrigerated and stored at the Kintampo Health Research Center and distributed on a weekly basis to CHOs where they will be stored at home in a cool place. This approach has been adopted to assure that the intervention is being conducted under conditions as similar as possible to those that would be found should the program be scaled up.

*Establishment of a data collection and monitoring system:* To assure that all data collection processes are in place and functioning prior to the beginning of recruitment into the study, the first month of the study will be focused on training CHOs, field workers and supervisors and testing the following data collection procedures: identification of households with pregnant women, the consent process, distribution of oxytocin in Uniject^TM^, storage of the oxytocin in Uniject^TM^ devices, blood loss measurement, completing the questionnaires, questionnaire transfer from the field to KHRC and data monitoring procedures and transfer procedures for women and/or newborns.

*Identification of households with pregnant women:* A large pool of community-based field workers with previous experience working on KHRC-sponsored community-based trials is available for employment by this study. It is anticipated that the study will require 75 field workers. Field workers will also be supervised by the nine field supervisors, for a Field Worker: Supervisor ratio of 8:1. The field workers must participate in and successfully complete a training program prior to commencement of the study. All study orientation activities will include the Ghana Health Service/ MOH policy message to deliver with a skilled attendant in a facility and to seek antenatal care. Activities to promote CHO assistance at birth will be targeted to women who cannot or do not choose to deliver in a facility. An information sheet describing the study and what each family would need to do to participate will be left with all households visited. Supervisors will monitor and document the number of pregnant women identified by each field worker and the gestational age of identified women on a monthly basis to determine if study assumptions are being met. Women in late pregnancy (at or around seven months gestation) will be invited to participate in the study *should they deliver at home.*  Informed consent to participate will be obtained (using full informed consent procedures. Those who accept will be asked to respond to a short interview to capture background characteristics, including asking participants if they know that whether they are carrying a multiple pregnancy. During the seventh through ninth months of pregnancy, the field worker will begin frequent surveillance of the pregnant woman’s household, with visits twice weekly and a visit by the CHO will be arranged.

*CHO assistance at birth:* Prior to commencement of the study, all CHOs who participate in the study will be required to complete a four to five day training course. Training materials will be developed by the Oxytocin Initiative team. Master trainers will be trained by the US-based team in collaboration with the in-country team. Training activities conducted by master trainers will be supervised by the in-country research team. CHOs must pass a competency-based exam specific to the cluster into which they are randomized before being permitted to participate in the study. A monitoring and evaluation system, including tools, has been developed to assure quality of study interventions. Following commencement of the study, families will contact the field worker by cell phone or other means at the onset of labor, and the field worker will then contact the CHO. The CHO will arrive by motorcycle or other means (using fuel provided by the District Health Management team, for which costs will have been covered by KHRC and the budget for this study). The CHO of the Field Worker, whoever arrives first, will reaffirm the woman’s consent to participate in the study, using a consent form specific to their cluster. CHOs in both groups will place a BRASSS-V calibrated drape designed to collect postpartum blood under the woman’s body before birth of the baby. Women will be asked to remain recumbent, if possible, for one hour if bleeding ceases within one hour, or within two hours if active bleeding continues at one hour. All blood collected in the drape will be scooped into the pouch, thus preventing the passage of blood at the back. The blood loss measure will exclude the fluid, urine and feces passed during the birthing process. Women will be cleaned using clean water and soap after the cessation of active bleeding. The drape will be removed from the woman and held up vertically in order to obtain the blood loss reading. This method has been validated and used in a number of previous and on-going trials.[16] The blood loss measurement will not interfere with the birth attendant’s management of the woman or newborn. The used study drape and blood will be disposed of in a latrine or other area suggested by the family.

It is very likely that none of the CHOs available to participate in this study will have had midwifery training, given that nationally only 4% of CHOs do. CHOs with midwifery training will not be eligible to participate in this study. The CHOs in both arms will be instructed not to manage the delivery. Only CHOs in the intervention group will administer oxytocin in Uniject^TM^ immediately following delivery of the baby. Intervention CHOs will also be trained to ask the woman if she is pregnant with twins (which will be done shortly after arrival at the household) and in all cases to palpate the uterus to detect the possibility of a twin prior to administering the injection of oxytocin. As described above, CHOs in both groups will be trained to recognize the causes for referral, to initiate referral for and to provide early treatment in the event of a postpartum hemorrhage. CHOs in the intervention arm will carry three Uniject packages to each delivery (one for one PPH prevention, one in the case of need for PPH early treatment and one for backup). CHOs in the control arm will carry two Uniject packages to each delivery. The used Uniject^TM^ devices will be disposed of in a sharps box provided by the study and returned to the study supervisors for audit purposes. The distribution of Uniject^TM^ devices to CHOs and their status will be monitored and documented throughout the study and results will be discussed with CHOs during weekly meetings. Unused Uniject^TM^ devices will be collected at the end of the study.

As a quality control measure, supervisors will monitor the time lapsed between calls to the CHO and their arrival at the household of study participants, based on CHO reporting of the timing of their activities. Supervisors will also monitor and document the completeness and errors in data collection forms completed by both CHOs and field workers. These results will be discussed in weekly meetings with the CHOs and field workers.

*Follow up interviews with recently delivered women:* Within 48-72 hours of delivery, the field worker will visit the homes of all women who gave initial consent to participate in the study. The questionnaire will be addressed first to the recently delivered woman enrolled in the study. However, completion of this interview, particularly for questions regarding the timing of administration of the oxytocin or other events during birth, may require input from a family member who was present at the delivery. The purpose of this follow up interview is to a) provide an assessment independent of the CHO regarding the timing of the administration of a uterotonic drug; and b) to document maternal and newborn outcomes which have occurred since the CHO left the household. In the event of an emergency referral at the time of birth, Field Supervisors will be responsible for visiting the hospital to collect information regarding the cause of the referral and the outcomes for woman and baby. At the initial consent, women will have agreed to have this information retrieved from the hospital in the event of an emergency referral. In the event of a maternal death, a family member present at the time of death will be asked consent to be interviewed. A Follow up questionnaire adapted for this purpose has been uploaded as part of this application. In the event of a maternal, fetal or early neonatal death, the internationally accepted WHO verbal autopsy questionnaires for women and infants will be used as an add-on to the Follow Up questionnaire.

*Follow up questionnaire for the non-family member, lay birth attendant:* Within 48-72 hours of delivery, the field worker will visit the non-family member birth attendant who was present at birth to administer a short questionnaire. The purpose of this interview is the same as for the follow up interview with recently delivered women. A consent form for the Follow Up interview specific to the traditional birth attendant is included in this application.

**Figure 1 Flow chart of procedures for the Oxytocin Initiative**

Randomization of 54 CHOs into intervention/control groups

Sensitization of the community

Identification of households with pregnant women

First visit by field worker, description of study and explanation of material included in the Study Information Sheet;

Follow-up visits by field worker to remind potential participants of the study and to encourage participation should the woman deliver at home. At 7^th^ month women are asked to participate in the study, are enrolled and informed consent is obtained. Consenting women are administered a short interview including questions on background characteristics, etc. Twice weekly visits are made until delivery.

Field Worker informed of delivery [either directly by the women, or by the TBA] and calls the CHO

CHO proceeds to delivery location, obtains final consent

Oxytocin in Uniject^TM^

n = 564

No uterotonic given

n = 564

Uterotonic administered or not and post-partum blood loss measured at one hour postpartum, or at two hours if there is on-going bleeding at one hour

Follow-up interview with woman/family member and Traditional Birth Attendant conducted within 48-72 hours by field worker to assess birth practices (and timing of injection) and outcomes

One month preparation period for study staff training and to test data collection procedures

1. **Number of study contacts:**

Most participants will have four study contacts: an initial visit when a pregnant women is identified and the study is explained to her and her family; a visit at seven months gestation when the field worker will request informed consent to participate in the study and will conduct a brief interview for those agreeing to participate; at delivery women will be visited by the CHO, where blood loss will be measured and the preventive intervention will be provided to women in the intervention arm of the study; at 48-72 hours following delivery, when all participants will be visited again by the field worker for the follow up interview, In addition, there will be a variable number of drop by visits during the seventh through ninth month of pregnancy to see if labor has commenced.

Table 2: Schedule of study visits

| **Type of Visit and Data Collected** | **Month of gestation** | | | **Day of delivery** | **Days following delivery** | | |
| --- | --- | --- | --- | --- | --- | --- | --- |
|  | **<7** | **7-8** | **9** |  | **1** | **2** | **3** |
| **Identification of household with a pregnant woman**: explanation of the study by the field worker using a Study Information Sheet which remains with the family. Woman’s name, address and gestational age are documented. | X |  |  |  |  |  |  |
| **Enrollment:** Pregnant women are asked to participate in the study should they deliver at home. Initial informed consent is requested/obtained. A short interview to document participant background characteristics, birth history is administered. |  | X |  |  |  |  |  |
| **Frequent (2x per week) drop by visits** by field worker to identify women in labor and to encourage women to participate in the study should they deliver at home. No data collection. |  | X | X |  |  |  |  |
| **In late pregnancy:** final informed consent for the injection (intervention group), and for blood loss measurement for all consenting women, completion of a questionnaire by the CHO regarding the birth including blood loss, administration of uterotonics or herbs by the family or CHO, complications, referrals, outcomes. |  |  |  | X |  |  |  |
| **Follow up interview:** by the field worker 48-72 hours following delivery. Interview includes documentation of procedures undertaken during and after delivery |  |  |  |  |  | X or | X |

1. **Expected duration of the study:**

The expected duration of the study, given the extension of data collection through November 2012, is 20 months. This includes a one month preparatory phase during which study staff are trained and data collection procedures are tested and refined; given the sample size re-estimation following the interim analysis, it is anticipated that 19 months of data collection will be required to obtain a sample of 1679 births for the comparative phase of the study. We are requesting approval up to 1850 women.

Stopping guidelines outlined in the DSMB charter are described for effectiveness, safety and futility. According to the original proposal: should preliminary trial results from the initial 50% of the sample meet the stopping guidelines for an effective intervention specified above, the three month post-comparative phase with universal distribution of the intervention to all CHOs will begin immediately (that is, at or around six months after the beginning of the study and following reporting to the Data Safety and Monitoring Board). Should preliminary trial results from the initial 50% of the targeted sample size meet stopping guidelines, suggesting a potentially harmful intervention, the trial will be stopped with no further data collection. If the preliminary analysis suggests inconclusive results, data collection will continue through nine months. The decision to implement the three month post-comparative universal distribution phase will be carried out at that point if the study hypothesis is supported by final results. The charter for the Data Safety and Monitoring Board has been uploaded as part of this application.

Following the interim analysis, the DSMB concluded that the conditional three month Universal Distribution Phase is not crucial and stated that from their perspective it is acceptable that investigators direct resources allocated for this activity to increasing the sample size of the comparative phase of the trial. Thus, in this amended study protocol, the sample size has been increased (see Section 3c) and the Universal Distribution Phase has been eliminated.

1. **Data analysis plan:**

The primary outcome for the study is postpartum hemorrhage, defined as 500ml or more blood loss within one hour after delivery of the baby, or within two hours if active bleeding persists at one hour. The risk of postpartum hemorrhage among deliveries to women assisted by intervention CHOs will be compared to the risk among deliveries managed by control CHOs, based on an intention to treat principle, adjusted for clustering. There are a number of secondary objectives and outcomes for this study for which results across intervention and control CHOs will be compared (the trial does not necessarily have sufficient statistical power to detect all these secondary outcomes). These include:

- 1. Safety: the % of deliveries during which the intervention and control CHOs administer oxytocin in Uniject *before* delivery of the baby.
  2. Safety: adverse maternal and fetal outcomes, including maternal deaths, stillbirths, early neonatal deaths, birth asphyxia; need for newborn resuscitation, uterine rupture; referral and/or transport to a higher-level facility. Comparisons will be made for all deliveries across both arms of the trial and restricted to deliveries for which oxytocin was given before delivery of the baby.
  3. Feasibility: The % of deliveries for which the CHO does not arrive at the pregnant woman’s house in time to intervene. The % of deliveries which occur after dark for which the CHO does not arrive in time to intervene. The average number of minutes required between the call to the CHO and the CHOs arrival at the woman’s house. The % of women who gave initial consent to participate in the study but who does not request CHO assistance at birth. The % of oxytocin in Uniject^TM^ devices that become unusable (according to the time/temperature monitor) over time when stored unrefrigerated, but in a cool place in the CHOs home. The % of oxytocin in Uniject^TM^ devices that are disposed of appropriately. The % of oxytocin in Uniject^TM^ devices distributed to CHOs that cannot be accounted for based on reported use. The reliability of CHO reporting of their own behaviors (the % of deliveries that show discrepancies between practices reported by the CHOs and by the woman/family member one week following delivery).

1. **Human biospecimen collection:**

Postpartum blood will be collected and measured using a calibrated drape designed for this purpose. Used drapes and blood will be disposed of in latrines or in other areas suggested by the family.

1. **Eligibility screening:**

Any woman at or around seven months gestation who agrees to participate in the study should she deliver at home is eligible to be enrolled in the study. Field workers will be responsible for assessing eligibility.

1. **Blinding:**

Given that the intervention CHOs will be administering prophylactic oxytocin in Uniject^TM^ following delivery of the baby, that control CHOs will not be administering prophylactic oxytocin, and that these differences will be visible to all present, blinding is not an option for this study.

1. **Routine care:**

CHOs in both arms of this trial will not manage deliveries of the women enrolled in the study. Women delivering in the presence of control CHOs will receive the same standard of care they would have received had they not participated in the study, with the important exception that emergency referral and transport can be assured if needed. Women delivering in the presence of the intervention CHOs will receive prophylactic administration of oxytocin in Uniject^TM^ during the third stage of labor, be assured of emergency referral and transport in the event of need, and will otherwise receive the same standard of care they would have received had they not participated in the study.

1. **Placebo or non-treatment:**

A placebo will not be used in this study.

1. **Treatment failure and participant removal criteria:**

Treatment failure is defined as an incident of postpartum hemorrhage following appropriately administered oxytocin in Uniject^TM^. Participant removal from the study would result from any change in eligibility. Such a change would most likely result from the need for referral to a higher level of medical care prior to administration of the intervention.

1. **Participant therapy at the end of the study:**

When the target sample size has been reached, CHOs will no longer be present for deliveries to administer oxytocin in Uniject^TM^, unless there are policy changes between now that would permit continued use of oxytocin by CHOs during the third stage of labor. If the results of the study demonstrate both community-level effectiveness in reducing rates of post-partum hemorrhage and an implementation profile that is favorable from safety and logistical viewpoints, the investigators will work closely with local and regional stakeholders to promote change in policy that will facilitate scaling up of the intervention.

1. **Participant referral:**

All CHOs will be trained to provide early treatment and initiate emergency referral in the event of postpartum hemorrhage (after reaching 500 mls blood loss). Early treatment includes administration of 10 IU of oxytocin in Uniject, fundal massage and emergency referral/transport to the nearest hospital. For referral, CHOs will contact KHRC via mobile telephone if a woman is still actively bleeding at 400mls blood loss. KHRC will have a vehicle and driver specifically available for this purpose who will immediately respond to the call. Should the car dedicated for this purpose be unavailable for some reason, KHRC will make another vehicle available. CHOs will accompany the woman to the hospital and remain there until the woman is admitted to the hospital. For each case of referral, supervisors will verify that referral procedures were followed and documented and any deviations from the protocol will be discussed with the CHO during weekly meetings with the supervisor. The CHO supervisor will be responsible for following up with hospital staff in order to document outcomes following the woman or newborn’s discharge from the hospital.

1. **Power calculations:**

Refer to Table 1 in #3c. The study was originally designed to detect a 50% decrease in the risk of postpartum hemorrhage (using the definition for PPH 1) with 80% power and a Type 1 error of 0.5%. Following the Interim Analysis, we re-estimated power for PPH1 and PPH2 (see Power Curves in Sample Size section).

1. **Reporting results to participants:**

There are no clinically relevant data collected that need to be returned to participants during the course of the study. However, we plan to disseminate overall results to Ghana’s Ministry of Health as well as Ministry of Health officials in Kintampo. We also plan to work with local and regional leaders to share the results with the community in an appropriate, context-specific manner.

**5. Data Security and Protection of Subject Confidentiality**

1. **We are not applying for a Certificate of Confidentiality**
2. **Security plan –** see below table.

| **Hard Copy of data collection form**: **Indicate your choice but typing an X in the appropriate box on the left:** | |
| --- | --- |
|  | Hard copies of data collection materials have identifiers and are locked in a secure cabinet or room with limited access by specified individuals. When possible, redacted (de-identified) versions of the data collection sheets will be used for coding and analysis. |
|  | Hard copies of data collection materials include an ID code and do not have personal identifiers. However, a code linking the data to the subject’s personal information is stored separately from the data collection sheets, and is locked in a secure cabinet or room with limited access by authorized individuals. |
|  | Data are not collected on paper. |
| X | Other (describe): All inhabitants in the four districts in which this study is being carried out have a KHRC identification number. This ID number will be recorded on Questionnaire #1 (Potential Participant Register) for each woman who is screened to participate in the study. The Potential Participant Register will remain in the Field Worker booklet (ie, it is not a loose leaf sheet of paper) throughout the duration of data collection. Once a week, selected variables (ID#, gestational age, estimated date of delivery, intended place of delivery and consent status) will be manually copied from the Potential Participant Register to Register #2 in the presence of Field Supervisors, who will then immediately submit these forms for data entry. All other data collection forms with personally identifying information have as the first page, a tear-off sheet with names, addresses and cell phone numbers. This sheet is referred to as the Label Sheet. These Label Sheets will be removed and destroyed once received and validated at KHRC.  All paper forms are collected from the field at the end of each week. The field supervisor will review the forms for completeness and submit, them to KHRC. The Data Coordinator for the study will scrutinize the forms prior to data entry and will have primary responsibility for assuring data quality. Data forms will be filed in secure cabinets at Kintampo Research Center. All staff involved in the study will have received training in the importance of confidentiality. For KHRC staff, maintaining confidentiality is a condition of hire/employment. |
| **Electronic Databases: Indicate your choice but typing an X in the appropriate box on the left: :**  *Note: A de-identified version of the database should be used for data analysis except in instances in which identifying information is prerequisite for coding or analysis. Databases that retain identifying information require a higher degree of electronic security.* | |
|  | The study is minimal risk and data collected are not sensitive in nature. No personal identifiers are included in the electronic database. Any electronic documents that link IDs to identifying information are stored on a computer in accordance with JHSPH Data Security guidance. |
| X | Personal identifiers are included in the database. The data are stored on a computer that is password protected with a secure server. Transfer or storage on portable devices (e.g., laptops, flashdrives) is encrypted. The devices on which this information is stored are accessible only to individuals who need access to these data. |
|  | No personal identifiers are included in the database but linkable identifiers exist separately and the data are sensitive in nature such that disclosure could provide a risk to the individual. The codes are stored on a computer that is password protected with a secure server. Transfer or storage on portable devices (e.g., laptops, flashdrives) is encrypted. The devices on which this information is stored are accessible only to individuals who need access to these data. |
|  | Other (describe): |

1. **Disposal of personal identifiers:** Hard copy data forms including the locator information form will be held in the Secure Archive Building at the Kintampo Health Research Center for a period of five years following the end of the data collection phase of the study.

**Destruction of data:**

Hardcopy data forms will be shredded five years following the end of the data collection phase of the study.

**6. Recruitment process:**

Recruitment in this study will be on the basis of face-to-face interactions between the pregnant woman,

her family members and study staff. As described in the section on study procedures, pregnant women

identified by field workers will have the study described to them verbally and will also receive a written

Study Information Sheet which will be left at the household. Later in pregnancy, potential participants

will be invited to enroll in the study and will be asked for initial consent. A specific consent to receive

the injection will be obtained for women in the intervention group at the time of delivery. Field workers

will request privacy when asking the woman for her consent to participate in the study. Participant

recruitment and informed consent will be carried out at three levels. First, community level meetings

will be organized with community opinion leaders including political leaders, religious leaders and

existing women’s groups. During these meetings, the study will be fully explained, including the risks

and benefits associated with participation. Community members will be allowed to ask questions and

seek clarifications on all issues. These community discussions will take place before the month-long

preparation phase of the trial. The remaining two levels of recruitment and consent are discussed in detail below.

**7. Consent process and documentation:**

Obtaining consent for individual level participation in the study will be handled in two steps: 1) an initial consent to participate in the study will be asked of all pregnant women at or around seven months gestation should they deliver at home; and 2) Following the woman’s request for CHO presence, a final consent to have blood loss measured (all study participants) and to have an oxytocin injection (intervention group participants only) will be reaffirmed once the CHO has arrived at the household for delivery. Initial and final consent forms are included in a separate file as part of this application.

The initial and final consent forms will be read aloud in Bono/Twi , the commonly spoken language of the pregnant woman. The potential participant will then be asked if she has any questions regarding participation in the study. Written, signed consent will be obtained with literate participants. When the consenting participant is not literate, a literate family member will serve as a witness. If there are no literate family members present, the Field Worker will either find a literate witness in the community or return at a later date when a literate witness will be available (for the initial consent). For the final consent, either the Field Worker or the CHO will find a literate witness in the community. Consent for women who do not read/write will be signed by the witness indicating that the consent was read to the woman and that she has agreed to participate. The participant will also be asked to provide her thumb print on the forms. Community field workers and CHOs will submit all signed consent forms to the Field Supervisor. All CHOs will be trained in the consent processes and the principles of international ethical guidelines for informed consent.  The CHOs are trained health care providers and, as such, are literate and familiar with the procedures being used during the study.

**8. Risks:**

*a. Risks:* Intramuscular administration of oxytocin, even in Uniject^TM^, requires skin puncture with a needle and therefore exposes the study participant to risks similar to those experienced when receiving any intramuscular injection: bruising, infection at the injection site, and slight pain/discomfort. In addition, CHOs may find it necessary to scoop blood into the pouch of the drape to prevent blood from passing out the back of the drape. For this and other reasons, all CHOs will wear gloves during this procedure.

*b. Minimizing the risks:* However, the Uniject^TM^ device is sterile by design and therefore the only risk of infection at the injection site results from unhygienic treatment of the affected surface area following the injection. Training for CHOs in the intervention arm will include when and how to use the oxytocin in Uniject^TM^ device, as a means of decreasing the slight risks to study participants from receiving an injection. Regarding risks to the CHOs, needle stick guidelines will be implemented based on national and state guidelines, and supplemented by WHO guidelines if necessary.

Women randomized to the control group will not receive prophylactic uterotonic drugs, and therefore may be at greater risk of experiencing postpartum hemorrhage than those in the intervention group. As current standard procedures for home births do not involve the administration of a uterotonic drug immediately following delivery of the baby, women randomized to the control group will not experience any increased risk as a result of participating in the study. However, both the intervention and the control group will offer: emergency referral and transport for any obstetric complication, as well as early treatment (injection of 10 IU of oxytocin in Uniject^TM^ and fundal massage) for cases of postpartum hemorrhage.

*c. Research burden for participants:* Study participants may be inconvenienced by the request to remain recumbent for one to two hours after delivery, however, they will not be restricted from getting up should they need to do so. The interview to be conducted at the woman’s home 48 to 72 hours following delivery will take less than 30 minutes to administer, and as such, does not pose a heavy burden on participants. There will be no out-of-pocket costs associated with participating in this study.

*d. Participant privacy during data collection:* None of the questions to be asked during enrollment, labor and delivery, or at the follow-up interview are of a highly sensitive nature. Regardless, the field worker will request that the follow up interviews with the recently delivered woman (and possibly a family member) and a traditional birth attendant who was present at the birth, be conducted in a private space.

**9. Benefits:**

Women who agree to participate in the study, regardless of the treatment group to which they are randomized, will receive early treatment in the event of postpartum hemorrhage. In addition, for women/newborns with complications during delivery or in the immediate postpartum period, CHOs will provide referral and arrange transport for the mother and/or newborn to the nearest health facility. Participants in the study will be insured by the study and thus have any research-related health care costs covered by this policy.

1. **Payment:**

There will be no compensation for mothers or their infants or households participating in this study.

1. **FDA regulated studies:**

Not applicable.

1. **Safety monitoring:**

An independent Data Safety and Monitoring Board (DSMB) will be established for this trial and will advise investigators on issues of safety as the study is implemented (The charter has been uploaded as part of the package of proposal materials). The DSMB will consist of five members: one biostatistician, three trialists (one of whom will be Ghanaian) and one obstetrician. Specific members include: Dennis Wallace, Senior Research Statistician, RTI International, Research Triangle, North Carolina; Robert Goldenberg, Professor, Drexel College of Medicine, Philadelphia, Pennsylvania; Jerry Costellano, Director, Institutional Review Board, Christiana Care, Wilmington, Delaware; Dr. Kohlkute, Director, National Institute for Research in Reproductive Health, Mumbai. India; Professor Agbenyega, University of Science and Technology, School of Medicine, Accra, Ghana. The DSMB will meet once prior to the start of data collection to review and approve the study protocol and the Manual of Field Operations. Thereafter, the DSMB will meet in-person or through teleconference as deemed appropriate by the members at approximately five months into the study. Approximately one month prior to these meetings, the dataset will be frozen for analysis. The DSMB will review a report summarizing results of preliminary analyses conducted by an external analyst ,and will decide whether to continue the study through the originally planned nine months or to stop the study. Recruitment and enrollment will continue in the field until a decision is reached. Stopping guidelines are described in the DSMB charter and are based on both the primary outcome and on safety as reflected by inappropriate timing of the use of oxytocin.

DSMB Update – February 27th, 2012: The DSMB met in December 2011 after 47% of the originally planned sample had been collected. After reviewing the data, the DSMB recommended continuation of the trial as planned, and suggested that any resources available should be redirected to extending the recruitment to the extent possible. A copy of the letter from the DSMB is included in the amendment application.

The DSMB may convene additional meetings (face-to-face or electronically) to discuss issues of safety and effectiveness of the intervention. At all meetings, minutes will be kept of the deliberations of the DSMB and made available to the IRB at the Johns Hopkins Bloomberg School of Public Health, PATH in Washington DC, the Kintampo Health Research Center in Kintampo, Ghana and the Ghana Health Service, Ministry of Health, in Accra, Ghana. Adverse event definitions will be agreed upon at the first DSMB meeting and all adverse events will be reported to the DSMB, and the IRBs at the Ghana Health Service, KHRC, PATH, Washington DC, the Johns Hopkins Bloomberg School of Public Health.

1. **Plan for reporting unanticipated problems/adverse events**:

Adverse event definitions for the purposes of reporting on human safety were agreed upon by the DSMB in April 2011. They include:

• Maternal death from any cause, with or without intervention;

• Uterine rupture, from any cause with or without intervention;

• Postpartum hemorrhage defined as 500 mL or more blood loss or referral for postpartum bleeding regardless of volume of blood loss;

• Administration of oxytocin prior to delivery of the baby;

• Stillbirth from any cause, with or without intervention;

• Early Neonatal Death (death before the Follow Up Interview which is within two to three days following birth) from any cause, with or without intervention;

• Needle stick by Community Health Officer, family member or anyone else associated with the study or present in the household at the time of birth;

The above adverse events will be reported by CHOs and Field Supervisors on the adverse event form and submitted to Dr. Sam Newton, the co-PI of the study who will finalize and sign the form. Dr. Newton will immediately forward this information on to the Ghana Safety Monitor and the DSMB for their review. Following PATH REC requirements, the Ghana Safety Monitor in collaboration with Dr. Sam Newton will only forward adverse event forms for review by the PATH REC and other concerned board for events which have been determined to be “unexpected, serious study related adverse events, disclosed events occurring at an unexpected frequency or unanticipated problems with the study which may pose a risk to the subjects or others. Other concerned boards include the Ghana Health Service and the Kintampo Health Research Center ethical review boards and the Johns Hopkins Bloomberg School of Public Health IRB. It is Dr. Newton’s responsibility to notify the IRBs within 72 hours of his initial notification of these adverse events. All adverse event forms forwarded to the IRBs will be encrypted.

Should previously unknown conditions in a participant be discovered as a result of study procedures, the participant will be advised to seek medical care. Regarding protocol deviations, the Data Management Center at KHRC will generate periodic reports to monitor the progress of the trial. Protocol deviations will be recorded and investigators will consider following up with individual CHOs where there is a pattern of non-compliance. Any unanticipated problems will be reported within 72 hours of their occurrence to the in-country Co-Principal Investigator, who will then contact the Ghanian Safety Monitor (the Ghanian member of the DSMB) and the IRBs at the Ghana Health Service, PATH and the JHSPH IRB. A JHU Problem Event form will be submitted immediately to the IRBs in the event of an unanticipated problem that results in the harm of a study participant.

1. **Other IRBs:**

This proposal has been reviewed by the Kintampo Health Research Center Institutional Ethics Committee in Kintampo, Ghana, Ghana Health Service Ethics Review Committee in Accra, Ghana, the PATH Research Ethics Committee in Seattle, Washington. The Ghana Food and Drugs Board in Accra, Ghana is a governmental regulatory body from which an approval letter is also required. A letter of approval from each of these institutions has been forwarded to the IRB at the Johns Hopkins Bloomberg School of Public Health.

1. **Outside collaborations:**

The Department of Population, Family and Reproductive Health at the Johns Hopkins Bloomberg School of Public Health has a formal agreement with PATH in Washington DC to carry out this study in collaboration with the Kintampo Health Research Center; KHRC has a formal agreement to collaborate with PATH, Washington DC, in which the Johns Hopkins Bloomberg School of Public Health is specifically cited as a technical partner involved in the study. PATH in Washington DC also has a signed Memo of Understanding with the Ghana Health Service outlining the research activities to be undertaken with the Kintampo Health Research Center. All institutions are supportive of the study and their role in it.

1. **Oversight plan for student studies:**

Not applicable.

1. **Oversight plan for studies conducted at non-JHSPH sites, including international venues, for which the JHSPH investigator is the responsible PI:**

This study will be conducted in the districts of Kintampo North, Kintampo South, Nkoranza North and Nkoranza South, in Ghana, a demographic surveillance site using the very well established infrastructure of the Kintampo Health Research Center, operating since 1994 and responsible for many large-scale, prospective clinical and community-based trials, with larger samples and more complicated designs than the Oxytocin Initiative. The study will be monitored on multiple levels. There will be weekly meetings between the the Field Supervisors and the community-based field workers, as well as the CHOs. There will be weekly meetings between the Field Supervisors and the co-PI on-site. Furthermore, the investigator team has both Ghanaian and Baltimore/Washington-based investigators with extensive experience conducting large scale field trials in low resource settings. In addition to the DSMB, and the Ghanaian Safety Monitor who review Adverse Event reports daily as they come in, the Steering Committee will have both scientific and administrative responsibility for the conduct of the Oxytocin Initiative trial. Adherence or changes to the protocol, assuring responsible financial and administrative reporting, reporting to the DSMB and decisions regarding publications, authorship, presentations, dissemination and data sharing all fall under the responsibilities of the Steering Committee. The membership of the Steering Committee will include, the Study Management Team that will include: Cynthia Stanton, PhD, Sam Newton MD, PhD, Luke Mullany, PhD, , Patience Cofie, MSc, PATH-Ghana, Sadaf Khan, MBBS, MPH, PHD. The Steering Committee will meet on a regular basis, as determined by the needs of the study. C. Stanton and/or L. Mullany have also scheduled at least two trips during data collection.

1. **Creation of a biospecimen repository:**

Not applicable.

**19. Data Coordinating Center:**

Not applicable.


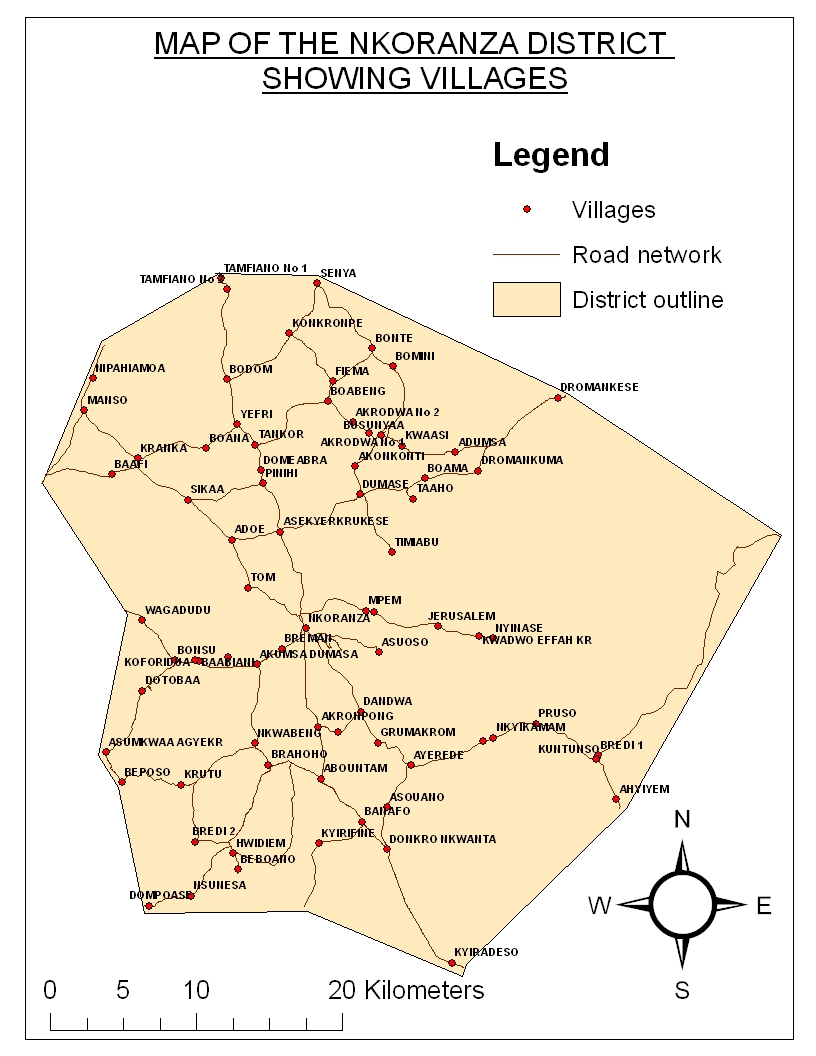
Appendix A: North and South Nkoranza Districts

Appendix B: North and South Kintampo Districts

**
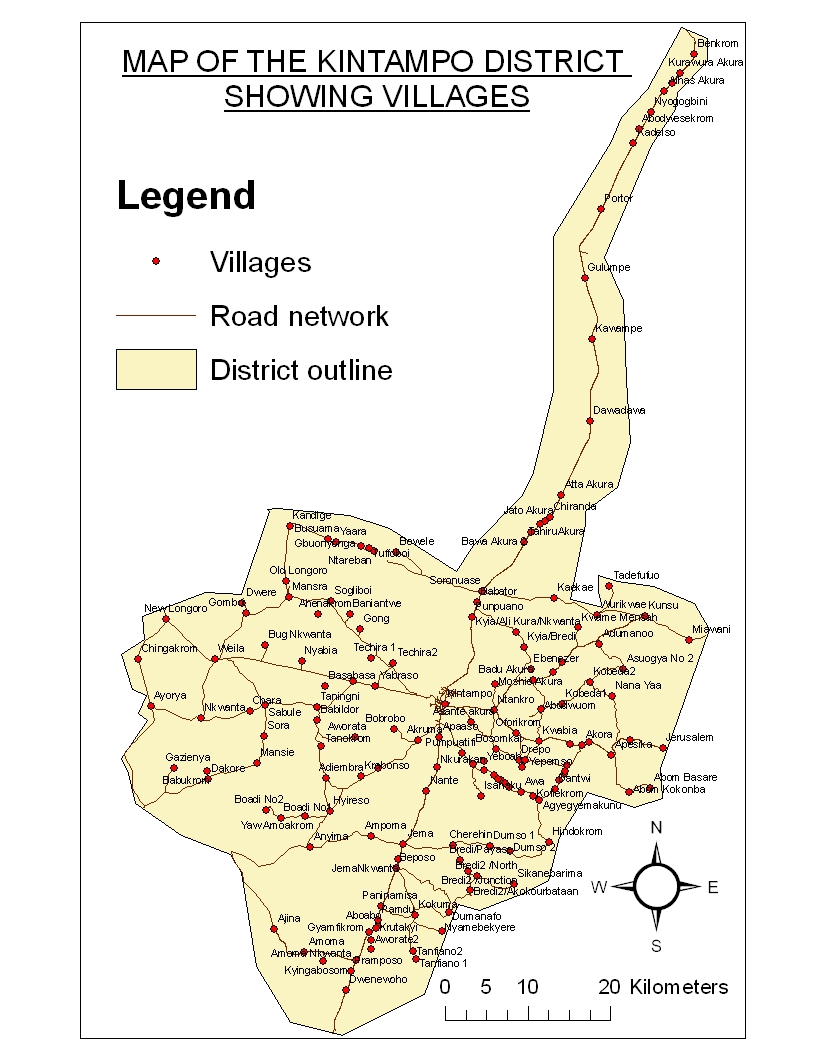
**

**References**

1. Prendiville, W.J., D. Elbourne, and S. McDonald, *Active versus expectant management in the third stage of labour.* Cochrane Database Syst Rev, 2000(2): p. CD000007.

2. Althabe, F., et al., *A behavioral intervention to improve obstetrical care.* N Engl J Med, 2008. **358**(18): p. 1929-40.

3. InternationalConfederationofMidwivesandInternationalFederationofGynaecologyandObstetrics, *Joint Statement, Management of the third stage of labour to prevent postpartum hemorrhage*. 2003: The Hague, London.

4. Mathai, M.G., A.M. et al.;, *WHO Recommendations for the Prevention of Postpartum Haemorrhage*. 2007, World Health Organization: Geneva.

5. Buekens, P., *Post-partum haemorrhage: beyond the confrontation between misoprostol and oxytocin.* The Lancet, 2010. **375**: p. 176-178.

6. Ghana Statistical Service, G.H.S.M.I., *Ghana Demographic and Health Survey, 2007*. 2009.

7. PATH. *Oxytocin in Uniject*. Technology Solutions for Global Health 2008 June 1, 2009 [cited.

8. PATH, *Oxytocin in Uniject*, in *Technology Solutions for Global Health*. 2008: Seattle, Washington.

9. PATH, *The Radically Simple Uniject Device: Rethinking the Needle to Improve Immunization*. 2009: Seattle, Washington.

10. Gulmezoglu, A.M.F., F;, *Prostaglandins for preventing postpartum hemorrhage.* Cochrane Database Syst Rev, 2007.

11. WHO, *Managing Complications in Pregnancy and Childbirth: A guide for midwives and doctors*. 2000, World Health Organization: Geneva.

12. Konje, J.C., O.A. Odukoya, and O.A. Ladipo, *Ruptured uterus in Ibadan--a twelve year review.* Int J Gynaecol Obstet, 1990. **32**(3): p. 207-13.

13. Zheng, Q.L.Z., X. M.;, *Analysis of 39 cases of maternal deaths caused by incorrect use of oxytocin.* Zhonghua Fu Chan Ke Za Zhi, 1994. **29**(5): p. 276-277.

14. Flandermeyer, D.S., C; Armbruster, D;, *Uterotonic Use in Home-births in Low Income Countries.* International Journal of Gynecology and Obstetrics, 2010. **published on line as of 1/20/2010**.

15. Derman, R.J., et al., *Oral misoprostol in preventing postpartum haemorrhage in resource-poor communities: a randomised controlled trial.* Lancet, 2006. **368**(9543): p. 1248-53.

16. Patel, A., et al., *Drape estimation vs. visual assessment for estimating postpartum hemorrhage.* Int J Gynaecol Obstet, 2006. **93**(3): p. 220-4.
